# Development and Implementation of Digital Diagnostic Algorithms for Neonatal Units in Zimbabwe and Malawi: Development and Usability Study

**DOI:** 10.2196/54274

**Published:** 2024-01-26

**Authors:** Hannah Gannon, Leyla Larsson, Simbarashe Chimhuya, Marcia Mangiza, Emma Wilson, Erin Kesler, Gwendoline Chimhini, Felicity Fitzgerald, Gloria Zailani, Caroline Crehan, Nushrat Khan, Tim Hull-Bailey, Yali Sassoon, Morris Baradza, Michelle Heys, Msandeni Chiume

**Affiliations:** 1 Population, Policy and Practice Institute of Child Health University College London London United Kingdom; 2 Biomedical Research and Training Institute Harare Zimbabwe; 3 Institute of Computational Biology, Computational Health Centre, Helmholtz Munich Germany; 4 Department of Child, Adolescent and Women’s Health, Faculty of Medicine and Health Science University of Zimbabwe Harare Zimbabwe; 5 Sally Mugabe Central Hospital Harare Zimbabwe; 6 Children's Hospital of Philadelphia Philidephia, PA United States; 7 Department of Infectious Disease Imperial College London London United Kingdom; 8 Kamuzu Central Hospital Lilongwe Malawi; 9 Snowplow Analytics London United Kingdom; 10 Baobab Web Services City of Cape Town South Africa

**Keywords:** mobile health, mHealth, neonatology, digital health, mobile apps, newborn, Malawi, Zimbabwe, usability, clinical decision support

## Abstract

**Background:**

Despite an increase in hospital-based deliveries, neonatal mortality remains high in low-resource settings. Due to limited laboratory diagnostics, there is significant reliance on clinical findings to inform diagnoses. Accurate, evidence-based identification and management of neonatal conditions could improve outcomes by standardizing care. This could be achieved through digital clinical decision support (CDS) tools. Neotree is a digital, quality improvement platform that incorporates CDS, aiming to improve neonatal care in low-resource health care facilities. Before this study, first-phase CDS development included developing and implementing neonatal resuscitation algorithms, creating initial versions of CDS to address a range of neonatal conditions, and a Delphi study to review key algorithms.

**Objective:**

This second-phase study aims to codevelop and implement neonatal digital CDS algorithms in Malawi and Zimbabwe.

**Methods:**

Overall, 11 diagnosis-specific web-based workshops with Zimbabwean, Malawian, and UK neonatal experts were conducted (August 2021 to April 2022) encompassing the following: (1) review of available evidence, (2) review of country-specific guidelines (*Essential Medicines List and Standard Treatment Guidelines* *for Zimbabwe* and *Care of the Infant and Newborn, Malawi*), and (3) identification of uncertainties within the literature for future studies. After agreement of clinical content, the algorithms were programmed into a test script, tested with the respective hospital’s health care professionals (HCPs), and refined according to their feedback. Once finalized, the algorithms were programmed into the Neotree software and implemented at the tertiary-level implementation sites: Sally Mugabe Central Hospital in Zimbabwe and Kamuzu Central Hospital in Malawi, in December 2021 and May 2022, respectively. In Zimbabwe, usability was evaluated through 2 usability workshops and usability questionnaires: Post-Study System Usability Questionnaire (PSSUQ) and System Usability Scale (SUS).

**Results:**

Overall, 11 evidence-based diagnostic and management algorithms were tailored to local resource availability. These refined algorithms were then integrated into Neotree. Where national management guidelines differed, country-specific guidelines were created. In total, 9 HCPs attended the usability workshops and completed the SUS, among whom 8 (89%) completed the PSSUQ. Both usability scores (SUS mean score 75.8 out of 100 [higher score is better]; PSSUQ overall score 2.28 out of 7 [lower score is better]) demonstrated high usability of the CDS function but highlighted issues around technical complexity, which continue to be addressed iteratively.

**Conclusions:**

This study describes the successful development and implementation of the only known neonatal CDS system, incorporated within a bedside data capture system with the ability to deliver up-to-date management guidelines, tailored to local resource availability. This study highlighted the importance of collaborative participatory design. Further implementation evaluation is planned to guide and inform the development of health system and program strategies to support newborn HCPs, with the ultimate goal of reducing preventable neonatal morbidity and mortality in low-resource settings.

## Introduction

### Background

In 2021, overall, 2.3 million children died in their first month of life, and the proportion of deaths below the age of 5 years attributed to neonates has risen from 40% in 1990 to 47% in 2021 [1]. The main causes of neonatal death include intrapartum-related complications and prematurity. Accurate, evidence-based identification and management of neonatal conditions could improve outcomes by standardizing care. It has been demonstrated in the literature that poor-quality care delivered by undertrained and overstretched health care professionals (HCPs) in low-resource settings can be attributed to approximately 60% of deaths from treatable conditions [2]. Clinical decision support (CDS) tools aim to standardize the assessment process and provide evidence-based guidance at the bedside, improving HCPs’ knowledge and competence. Previous studies have demonstrated that, owing to limited laboratory diagnostic capabilities, within these low-resource settings, there is significant reliance on clinical findings to make a diagnosis [2-4]. The implementation of diagnostic pathways could improve outcomes for this vulnerable neonatal population [4]. In addition, the development of management pathways adaptable and modifiable to resource availability is paramount [5]. There is potential for digital CDS tools to support HCPs to deliver improved neonatal care despite resource limitations and hence optimize outcomes.

The use of decision support tools has been well established and supported in health systems globally [6]. In this digital age, mobile phone coverage and digital uptake in low-resource settings has surged. Together with the low-dose, high-frequency education strategies [7,8] to improve newborn care, digital interventions appear to present a promising approach to improving newborn care [9,10].

Digital CDS tools are designed to aid directly in clinical decision-making and can use individualized patient data to guide management to implement evidence-based clinical guidelines at the bedside [11]. Paper-based guidelines are established resources, but these often get lost, are placed on shelves, and become easily outdated and therefore redundant. The systematic review of CDS systems by Kawamoto et al [12] identified four features for improved clinical practice:

1. Automatic provision of decision support as part of clinician workflow
2. Provision of recommendations rather than just assessments
3. Provision of decision support at the time and location of decision-making
4. Computer-based decision support

To date, most digital tools have implemented CDS as stand-alone tools to provide additional support to clinicians [9,13]. Consequently, the use of these tools can be limited as their use is voluntary and assumes clinician readiness and preparedness to adopt digital tools for which they may not have preexisting training or knowledge [14]. There is a growing evidence base for newborn digital interventions in low-resource settings ranging from patient level (NeMo tool trialed in Uganda, designed to support mothers in identifying newborns who are sick [15]) to system level (such as the World Health Organization standards-based, machine-readable, adaptive, requirements-based, and testable guidelines) for systematic pathways for the development and implementation of localized digital systems [16]. Digital interventions have the potential to both improve neonatal quality of care in low-resource settings and, in turn, improve neonatal outcomes, but there is a growing need for these interventions to be rigorously evaluated and to move from pilot or small-scale projects to scale [17]. Key elements to optimize the generalizability and scalability of such interventions are the vital importance of software sharing and the ability to have the intervention open source, which offers lower costs; greater transparency; faster iterations; and importantly, for the low-resource setting, local ownership in comparison with proprietary and, often siloed, digital interventions.

### Neotree

Neotree was developed as an integrated, open-source, digital, quality improvement system for hospital-based sick and vulnerable newborns [18-20]. The evidence suggests that up to 70% of newborn deaths could be prevented by the implementation of evidence-based interventions [21]. The system guides HCPs through the admission and clinical care of newborns. It combines evidence-based clinical guidelines with real-time newborn data collection, data visualization, data export, and newborn education on a single platform [22]. This tablet-based digital system is for use at the hospital bedside by HCPs with a range of skills and competencies supporting the care and treatment of newborns. It is currently implemented in the neonatal units at Sally Mugabe Central Hospital (SMCH) and Chinhoyi Provincial Hospital in Zimbabwe and at Kamuzu Central Hospital (KCH) in Malawi. SMCH’s neonatal unit is a physician-led unit with mainly junior physicians (1-2 years after graduating) using Neotree and providing neonatal care, with consultant oversight; Chinhoyi Provincial Hospital and KCH are both nurse-led or midwife-led units with consultant oversight. Neotree offers a solution that integrates directly into everyday care for all admitted newborns. Crucially, the CDS functionality is not dependent on nurses or clinicians having any specialist knowledge about the care of the newborn. This integration and development process is consistent with the suggestions by Kawamoto et al [12]. An overview of the development and implementation experience so far can be found elsewhere [18,22]. To date, Neotree has aided in the care of approximately 35,000 newborns and the education and training of >1000 HCPs at the implementation sites in Malawi and Zimbabwe and has demonstrated high acceptability, feasibility, and usability with perceived and observed improvements in newborn care [18,22].

### The CDS Function Development

The aim of the CDS function was to ultimately provide a personalized, integrated, evidence-based management plan (with embedded educational messaging around newborn care), tailored to the health care facility’s workflow and resource availability. In addition, a list of potential clinical problems was provided to facilitate pattern recognition and for education purposes. Evidence-based algorithms were precoded into Neotree management guidelines for the programmed conditions. Therefore, the entered data instantaneously provide a list of potential clinical problems and associated clinical management actions at the bedside.

The algorithms fell into four categories:

1. *Simple condition-based decision trees based on good evidence* (eg, prematurity or low birth weight or resuscitation)
2. *Simple conditional expressions based on weak evidence* and best clinical judgment (eg, jaundice)—these require additional refinement with evidence review and clinical consensus
3. *Complex conditional expressions based on good evidence* (eg, Thompson score for neonatal encephalopathy)
4. *Complex conditional expressions with weak evidence base* (eg, sepsis)—these require analytical refinement of the algorithm and testing

### Phase 1

First-phase CDS development in Zomba Central Hospital, Malawi, included developing and implementing neonatal resuscitation and stabilization algorithms, conducting a Delphi study to review key algorithms (neonatal sepsis, hypoxic ischemic encephalopathy, respiratory distress of the newborn, and hypothermia) [23], and developing the initial versions of CDS to address a range of neonatal conditions [20]. The resuscitation and stabilization algorithms were configured in 2016 based on the World Health Organization guidelines and Helping Babies Breathe and have been activated throughout implementation, so that, for example, if a baby is not breathing, Neotree will advise how to resuscitate [20]. Clinical management support pages were also configured for several potential diagnoses. These appeared in Neotree at the end of the admission process according to the HCPs’ chosen diagnosis and included hypoxic ischemic encephalopathy or birth asphyxia, prematurity, neonatal sepsis, gastroschisis, and transient tachypnoea of the newborn. The development of these clinical management support pages continued through 2018 and 2019. Neotree uses a web-based editor function using “variable expressions” to describe the condition, which is coding depicted by a “$” and brackets (‘’). The paper by Khan et al [22] describing the software development of Neotree describes the web editor function in detail; an example can be found in Multimedia Appendix 1.

Evaluating solutions such as CDS is important for both improvement and assessment of the effectiveness of the app [24]. For Neotree, evaluation and user feedback has been collected in three distinct ways: (1) on an ongoing basis, (2) during usability workshops, and (3) through questionnaire evaluation [20,25].

### Phase 2

This study aimed to conduct the second phase of the algorithm development. In addition, we aimed to understand the user experience of the CDS functionality. As integrating such tools in routine care is not part of standard practice, we hoped to understand the strengths and weaknesses of these solutions and how we can optimize the implementation of such solutions in the future.

## Methods

### Overview

**Figure 1:**
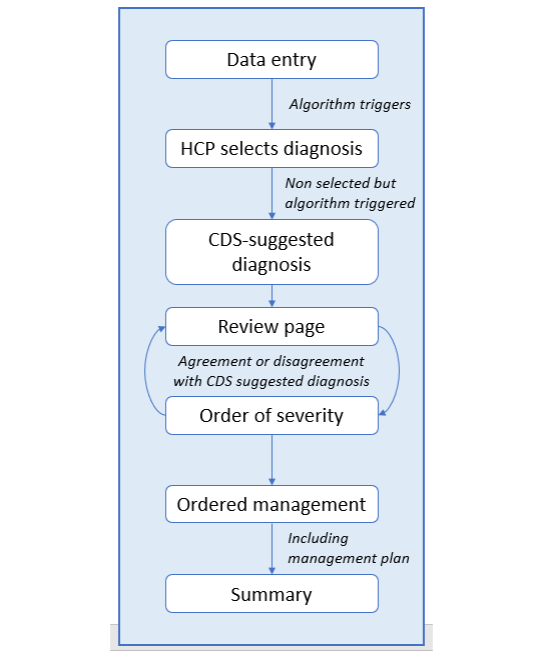
Overview of the clinical decision support (CDS) pipeline in Neotree. HCP: health care professional.

The second phase of CDS development was to integrate the clinical data entered during admission to trigger evidence-based diagnostic algorithms alongside personalized management guidelines, tailored to health care facility’s resource availability. On the basis of the clinical data entered by the HCP using Neotree, nested algorithms would determine whether a diagnosis should be triggered and, if so, would provide a suggested diagnosis, as shown in Figure 1.

**Figure 2:**
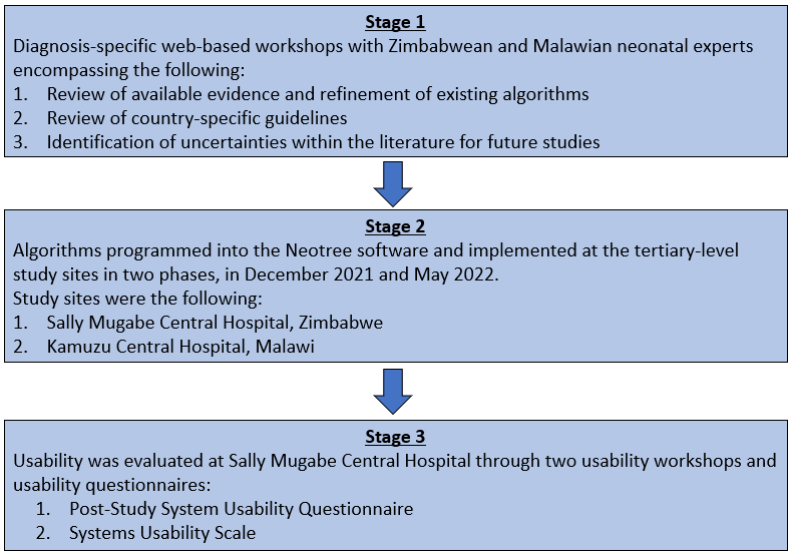
Phase-2 clinical decision support development process (3 stages).

First, it allows the HCPs to select their own diagnoses from a list of differentials. It then provides all the algorithmic diagnoses generated from clinical data (but not selected by the HCP) as “suggested diagnoses.” Phase-2 development of the CDS algorithms was planned in 3 stages (Figure 2).

### Stage 1

To develop the algorithms required to implement CDS, a team of subject matter experts was formed and comprised of neonatal clinicians based in Zimbabwe, Malawi, and the United Kingdom. The experts were selected based on their extensive experience in and knowledge about delivering newborn care within the settings and the limitations within their environment and context; the UK-based experts had both neonatal experience and detailed knowledge about the Neotree system. Importantly, the Zimbabwean and Malawian experts had detailed knowledge of resource and equipment availability within each setting, which ensured the development of actionable management guidance. Web-based workshops were conducted to discuss each clinical diagnosis. All clinical problems and potential diagnoses were described clearly under the following headings:

1. *Measurement* (how and when)
2. *Categorization* (with reference to evidence)
3. *Variable expression* (how the diagnosis is or will be coded within Neotree)
4. *Clinical management advice* (with reference to evidence)
5. *Needs for further refinement* (next steps required)

The algorithm triggers were clearly defined and agreed upon, and subsequent clinical management was based on local, national, and international clinical guidelines and adapted based on local resource availability. Textbox 1 shows an example of the discussions, and “$” denotes the variable being described. The national guidelines used were the *Essential Medicines List and Standard Treatment Guidelines for Zimbabwe* [26] and *Care of the Infant and Newborn, Malawi*.

Mock clinical test cases were created by the expert team and were used for active testing in Neotree to ensure that the algorithms were triggered in the correct circumstances and were intuitive to use, leading to further refinement. This testing was performed by Neotree team members and then a cohort of HCPs who had been trained and used Neotree in their everyday clinical practice.

**Example of stage-1 discussions—thermoregulation (“$” denotes the variable being described).**

**Example**

- *Measured or recorded*—based on temperature recorded on admission
- *Categorization—* according to the World Health Organization guidelines, there are 5 possible categories for temperature on admission:
  Mild hypothermia: temperature 36°C-36.4°C
  Moderate hypothermia: 32°C-35.9°C
  Severe hypothermia: <32°C
  Normothermia: 36.5°C-37.5°C
  Hyperthermia: >37.5°C
- *Variable expression* ($ denotes the variable being described)
  Algorithm version 2: proposed from the workshop
    - Mild hypothermia: $temperature >35.9°C and $temperature <36.5°C
    - Moderate hypothermia: $temperature >31.9°C and $temperature <36°C
    - Severe hypothermia: $temperature <32°C

### Stage 2

Following the finalization of the individual algorithm pathways, the CDS functionality was deployed in 2 cycles (December 2021 and May 2022). The first cycle included the first set of clinical diagnoses (update to resuscitation and stabilization, thermoregulation, convulsions, low birth weight, prematurity, hypoglycemia, HIV, and respiratory distress), and the second cycle comprised clinical diagnoses that required more extensive review and definition by the expert team (neonatal encephalopathy; sepsis; jaundice; and congenital abnormalities, which include cleft lip or palate, congenital dislocation of the hip, talipes, gastroschisis, omphalocele, and spina bifida). These were to be deployed only in the 2 central hospitals (SMCH in Zimbabwe and KCH in Malawi), as both centers have senior neonatal clinical oversight.

### Stage 3

Usability evaluation was planned to include usability workshops and usability questionnaires.

The usability workshops were conducted to understand the user experience and to provide specific information about the steps and items that require improvement in CDS design and development. The workshops were conducted using a think-aloud approach [27]. The HCPs, familiar with Neotree, were provided with a specific medical scenario (designed by the expert team) and asked to complete a Neotree admission in real time. These workshops were conducted in a nonneonatal environment to allow users to focus on their experience and understanding of the app instead of having to focus on neonatal clinical care. The provided scenario simulated a typical admission. Interviews were conducted by the interviewer in English (the clinical working language of both Zimbabwe and Malawi). The interview was recorded and later transcribed by the interviewer. While the user thought out loud and provided feedback about the app, the interviewer took short notes to prompt further questioning, especially about the CDS functionality.

Usability questionnaires such as the System Usability Scale (SUS) and the Post-Study System Usability Questionnaire (PSSUQ) provide additional quantifiable insights and metrics into the user experience of CDS [28]. PSSUQ assesses 3 categories: system usefulness, information quality, and interface quality. These 3 domains cover 16 questions, rated on a Likert scale ranging from 1 (strongly agree) to 7 (strongly disagree). Participants could also indicate if the question was not applicable to them. The SUS is a 10-item Likert scale questionnaire with options ranging from 1 (strongly disagree) to 5 (strongly agree). The SUS score was compared with an internationally recognized measure of acceptable usability of 68 out of 100. Both survey questionnaires were chosen because of their popularity in assessing mobile health solutions in low-resource settings. For the quantitative measurements (SUS and PSSUQ), means and SDs were calculated using Stata (version 17.1).

### Ethical Considerations

The Neotree study, including the wider implementation evaluation of Neotree and the CDS functionality development, received approval from SMCH Research ethics committee (reference HCHEC070618/58), University College London ethics committee (5019/004), Biomedical Research and Training Institute (AP148/18), Medical Research Council of Zimbabwe (MRCZ/A/2570), and Electronic Health Records Department of the Zimbabwe Ministry of Health and Child Care. The collection of qualitative data from HCPs using Neotree to assess the usability of all functionalities was also approved. Written informed consent was obtained from all usability evaluation participants, and their results were deidentified and stored anonymously. Attendance at the workshops was compensated through reimbursement of travel costs and refreshments. Participants were reimbursed US $10 for their time and travel costs as per the Zimbabwean Medical Research Council policy.

## Results

### Algorithm Development

Overall, 11 diagnosis-specific web-based workshops with Zimbabwean and Malawian neonatal experts were conducted between August 2021 and April 2022, encompassing the following:

1. Review of available evidence
2. Review of country-specific guidelines (Essential Medicines List and Standard Treatment Guidelines for Zimbabwe and Care of the Infant and Newborn for Malawi)
3. Identification of uncertainties within the literature for future studies

Table 1 shows the diagnoses discussed and the phase they were introduced in.

**Table 1:** Diagnosis-specific algorithms and their introduction (cycle 1 and cycle 2).

Data source and diagnosis		Cycle 1 introduction (December 2021)	Cycle 2 introduction (May 2022)
**Simple conditional-based decision trees based on strong evidence**			
	Low birth weight	✓	
	Prematurity	✓	
	HIV exposure	✓	
	Thermoregulation	✓	
	Hypoglycemia	✓	
**Simple conditional expressions based on weak evidence**			
	Jaundice		✓
	Convulsions	✓	
**Complex conditional expressions based on strong evidence**			
	Neonatal encephalopathy		✓
	Respiratory distress	✓	
**Complex conditional expressions based on weak evidence (in low-resource settings)**			
	Sepsis		✓
	Congenital abnormalities		✓

Detailed discussions and clinical triggers, documented in the A to E format (discussed previously), alongside the country-specific management plans and the coding used for Neotree can be found in Multimedia Appendix 2 [23,26,29-35]. Where national management guidelines differed, for example, birth weight thresholds for admissions to the neonatal unit, country-specific guidelines were created. Management guidelines were created based on resource and equipment availability in each setting. These international meetings also facilitated cross-site learning and quality improvement discussions. The clinical content and Neotree algorithm coding for each developed algorithm can be found in Multimedia Appendix 2.

The first-cycle algorithms developed, including low birth weight, convulsions, and hypoglycemia, had internationally defined descriptions and management guidelines, with minimal variation between nationally agreed management pathways. These diagnoses were relatively straightforward to discuss among the experts and gain unanimous agreement on the clinical content.

The more complex diagnoses proved to be more challenging and required multiple meetings with the experts to be clearly defined. As a result, neonatal encephalopathy and neonatal sepsis were assigned to ongoing international working groups for continuing research. The respiratory distress algorithm was split into term (defined as ≥37 weeks of gestation) respiratory distress and preterm (defined as <37 weeks of gestation) respiratory distress, and we identified the need for a more formal review of the literature within the global health context.

After agreement of clinical content, the algorithms were programmed into a test script, tested with the respective hospital’s HCPs, and refined according to their feedback.

**Figure 3:**
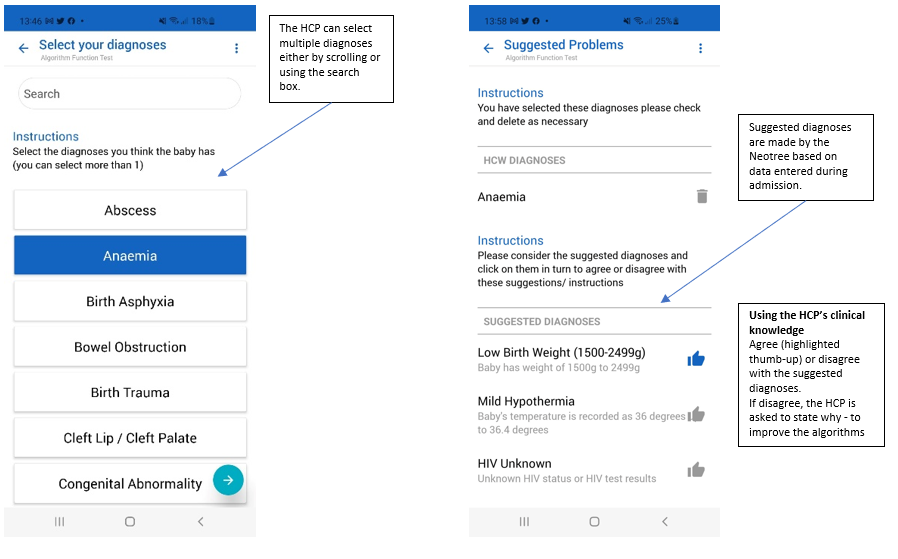
Algorithm-derived diagnosis page in Neotree. HCP: health care professional.

Figure 3 shows how the algorithm-derived diagnoses are presented within Neotree.

### Usability Workshop Results

#### Overview

**Figure 4:**
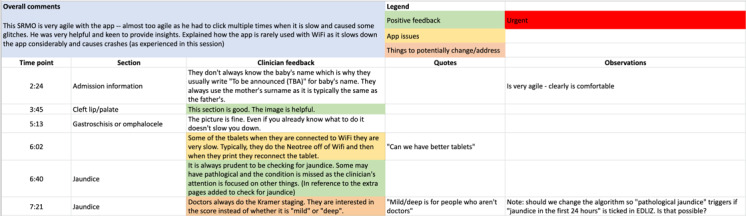
Excerpt of records from a usability workshop conducted after the release of the second set of clinical decision support, in May 2022. EDLIZ: Essential Medicines List and Standard Treatment Guidelines for Zimbabwe. SRMO: senior resident medical officer.

In total, 2 workshops were conducted in both Zimbabwe and Malawi after the release of each CDS—in January 2022 and in June 2022. An example of the recorded feedback is shown in Figure 4*.* In Zimbabwe, the workshops were attended by 5 HCPs—2 (40%) female participants and 3 (60%) male participants. In Malawi, the workshops were attended by 4 HCPs—3 (75%) female participants and 1 (25%) male participant, and feedback was recorded.

From the usability workshops that were conducted, three main themes emerged, two of which related directly to CDS, and the last one related to the Neotree tablet:

1. Education
2. Streamlining of processes
3. Technical challenges

#### Education

The main theme prevailing across interviews was the positive feedback toward the availability of information and clinical guidance provided through Neotree and CDS. As most of the clinicians using Neotree are junior physicians, especially at SMCH, they have limited experience in the management of neonates:

This might seem obvious but on your first day it is not.

Participant 2

#### Streamlining of Processes

HCPs highlighted that CDS in Neotree was found to streamline and systematize clinical diagnosis and management at the hospital, resulting in better and more effective care. This was done by implementing diagnostic algorithms that provide data-driven results and by providing management for the clinical diagnoses that are based on the most up-to-date guidelines. By providing the management pages for each diagnosis (and a repertoire of all management pages) in a centralized location, Neotree provides streamlined care for newborns as clinicians do not resort to a multitude of different resources that may not agree in management:

These are the nevirapine doses? You have outdone yourself. I like this very much because we learn about HIV guidelines but sometimes you just forget so this is nice.

Participant 5

Despite the ability of Neotree to provide data capture, CDS, and clinical management, this proved to extend the admission process. Clinicians highlighted the duration of admission completion to be a problem, especially in an already overstretched and understaffed hospital:

Sometimes when you’re on call you don’t have time to go through it.

Participant 2

Takes a lot of time when you have 6 babies screaming.

Participant 4

Admitting a patient really does take 15-20 minutes.

Participant 1

#### Technical Challenges

During the workshops, the tablets had issues with general degradation as they had just reached 3 years of use in the hospitals. These challenges resulted in the app crashing or not reacting to user input. The users, however, did not show frustration and mentioned that because it happens in the ward, they have grown used to it and have adapted to the challenges. Despite the clinicians adapting to these issues, this is severely affecting their experience of using the tablets and CDS, which may provide explanation for the relatively low scores in the SUS and PSSUQ questionnaires with respect to technical capability:

Can we have better tablets.

Participant 2

Just delete the ones that clicked themselves here.

Participant 4

I guess I’ll wait for it.

Participant 4

### Usability Questionnaire Results

In this study, usability of the Neotree CDS was also assessed using the PSSUQ (8/9, 89%) and SUS (9/9, 100%) questionnaire at SMCH.

The same group of HCPs was asked to complete 2 questionnaires (SUS and PSSUQ). One person failed to complete the PSSUQ due to time constraints on the neonatal ward. The SUS results are shown in Table 2, and the PSSUQ results are shown in Table 3. SUS showed generally high usability of the CDS system in Neotree, especially relating to aspects such as desire to use the system (questions 1, 7, and 9) and ease of use (questions 3 and 7). In contrast, the CDS system scored low on questions relating to technical complexity and difficulties (questions 2, 4, 6, 8, and 10).

The PSSUQ showed results similar to those of the SUS, where the technical complexities of the system (question 8) was highlighted as an issue.

Overall, both usability scores (SUS mean score 73.8 out of 100 [higher score is better]; PSSUQ overall score 2.28 out of 7 [lower score is better]) demonstrated high usability of the CDS function (comparable with previous SUS scores assessing the usability of Neotree data capture and dashboard functionalities in Malawi [mean score 88.1 out of 100] [25]) but highlighted issues around technical complexity, which have been subsequently addressed.

**Table 2:** System Usability Scale (SUS) results (N=9)^a,b^.

Questions	Score, mean (SD)
1. I think I would like to use this system frequently	4 (1.4)
2. I found the system unnecessarily complex	2 (1.5)
3. I thought the system was easy to use	3.9 (1.4)
4. I think that I would need the support of a technical person to be able to use this system	2.1 (0.9)
5. I found the various functions in this system were well-integrated	3.7 (1.4)
6. I thought there was too much inconsistency in this system	1.9 (0.6)
7. I would imagine that most people would learn to use this system	3.9 (1.4)
8. I found the system very cumbersome to use	1.8 (1.1)
9. I felt very confident using the system	4.4 (1.1)
10. I needed to learn a lot of things before I could get going with this system	1.8 (0.8)

^a^Questions were answered using a Likert scale ranging from 1-5, where 1=strongly disagree and 5=strongly agree.

^b^Calculated SUS score (total converted mean scores × 2.5)=73.8.

**Table 3:** Post-Study System Usability Questionnaire (PSSUQ) results (n=8)^a,b^.

Questions	Score, mean (SD)
1. It was simple to use this system	1.9 (1.7)
2. I was able to complete the tasks and scenarios quickly using this system	1.9 (1.7)
3. I felt comfortable using this system	2.3 (1.6)
4. It was easy to learn to use this system	2 (1.7)
5. I believe I could become productive quickly using this system	1.8 (1.8)
6. The system gave error messages that clearly told me how to fix problems	2.1 (1.4)
7. Whenever I made a mistake using the system, I could recover easily and quickly	2.3 (0.9)
8. The information (such as online help, on-screen messages, and other documentation) provided with this system was clear	4.1 (2)
9. I needed to learn a lot of things before I could get going with this system	2.5 (1.6)
10. It was easy to find the information I needed	2.6 (1.5)
11. The information was effective in helping me complete the tasks and scenarios	2.1 (1.4)
12. The organisation of information on the system screens was clear	1.9 (1.7)
13. The interface of this system was pleasant	1.6 (0.5)
14. I liked using the interface of this system	1.6 (0.7)
15. This system has all the functions and capabilities I expect it to have	3 (1.3)
16. Overall, I am satisfied with this system	2 (1.3)

^a^Questions were answered using a Likert scale ranging from 1-7, where 1=strongly agree and 7=strongly disagree.

^b^Calculated total PSSUQ score=2.28.

## Discussion

### Principal Findings

This study describes the successful development and pilot implementation of the only known neonatal CDS system incorporated within a bedside data capture system with the ability to deliver up-to-date management guidelines, tailored to local resource availability. Bucher et al [9], Mitchell et al [36], and Long et al [17], as examples, describe CDS digital tools generally designed for the pediatric population, not specifically neonatal, and without incorporated digital data capture. To date and to the best of the authors knowledge, there is no other neonatal digital intervention designed to improve neonatal care using these methods. As a significant proportion of the global mortality below the age of 5 years is attributed to neonatal deaths in low-resource settings, the development of sustainable interventions targeting this vulnerable group are vital. The incorporated real-time usability evaluation demonstrated high usability from HCPs actively using Neotree in their everyday practice, within 2 low-resource settings—Zimbabwe and Malawi. The adaptability of the Neotree system allowed for real-time changes to the app to improve the processing times and algorithm function. This enabled changing the surfacing format of the algorithm-selected diagnoses to improve the usability of the CDS function.

It has been well documented in the literature that improving adherence to evidence-based clinical guidelines can improve the quality of care and, in turn, patient outcomes. However, this has been challenging to effectively implement, particularly in low-resource settings [37,38]. A multifaceted approach is required, and a system with the ability to adapt is therefore essential, both to resource availability and guideline updates. With the plethora of digital health interventions implemented in low-resource settings, centralizing information for HCPs instead of relying on memory, wall charts, or printed guidelines has been demonstrated to improve adherence [36]. The target product profile developed by Pelle et al [37], for electronic CDS algorithms in low-resource settings, sets out specific guidance about the development of such interventions. The algorithms need to be based on evidenced clinical guidelines and be customizable to the context. This has been heavily incorporated into the design and methodology of the development of the Neotree CDS functionality.

Comparison can be made with studies within the pediatric population. Several studies have reviewed the feasibility of the implementation of electronic, user-friendly Integrated Management of Childhood Illness tools, for example, in both Tanzania and South Africa [36,39]. In the Tanzanian setting, adherence to guidelines was demonstrated to improve the use of electronic versus paper-based systems [36]. In comparison, the South African study demonstrated the challenges with technical issues and workload implications [39]. Technical challenges and workload implications were also highlighted in the usability workshops for Neotree; however, despite these challenges, the system scored well on the usability questionnaires. The HCPs stated that they had adapted to these challenges.

Detailed understanding of the context and setting within which the digital intervention has been implemented is an essential component for sustainability. Many of the comments from the interviewed HCPs described the understaffed, demanding, and poorly resourced environment they work within, which has been described in the literature and is common across many health institutions in low-resource settings [40]. Understanding this context and the complexities of adding a digital intervention, such as power failures within degrading infrastructures or additional workload, is key in successful implementation [17]. Alongside this aspect is the importance of collaborative participatory design. Many health care interventions have been developed from the high income–setting perspective with minimal attention to the real-life adaptation within low-resource health care settings. Collaborative participatory design and research can help to close the gap between research, policy, and practice [41]. The contextual reality that many low-resource settings face with frequent crises or shocks, such as economic crises, natural disasters, or health crises, inevitably has an impact on how well the interventions are implemented. Damschroder et al [42] have stated that “Many intervention studies have been found to be effective in health services research but fail to translate into meaningful patient care outcomes across multiple contexts.” The Neotree algorithm workshops including Zimbabwean and Malawian experts demonstrated the key importance of these international relationships for the CDS algorithms to be usable within their intended context. The customizability of the CDS algorithms to resource availability and the perceived streamlining of processes from diagnosis to management increased the usability of Neotree by incorporating the detailed knowledge about the working environment.

### Limitations

The introduction of the algorithms had a perceived impact on the duration of the admission process. In an already overburdened neonatal unit, the increased time of use could have implications on both its acceptability and use. However, the time-use analysis and cost analysis (paper in progress) by Haghparast-Bidgoli et al [43] found that admission times were similar when comparing Neotree with paper-based systems. Implementation in low-resource settings is always going to be influenced by the environment and context it is used in. In Zimbabwe, a degrading health care system [40] poses challenges to any digital tool to be implemented in this setting. The usability questionnaires were only performed in SMCH in Zimbabwe. As SMCH is a predominantly physician-led unit, comparative Malawian and Zimbabwean CDS functionality qualitative data are needed to ensure that usability evaluation across all HCP cadres is reviewed; more comprehensive usability data are required and therefore planned to be collected.

### Next Steps

Further usability evaluation is planned 1 year after implementation as part of the wider sustainability program of Neotree. This will again include the usability workshops and questionnaires with real-world observation, similar to previous usability testing of the data capture functionality. Approvals from the Pediatric Associations of Zimbabwe and Malawi are pending. The assigned international working groups for continuing research are ongoing. The Neonatal Sepsis Working Group is currently in the process of an in-depth review of neonatal sepsis guideline use within low-resource settings [44], conducting a scoping review [45], and developing a robust clinical prediction model with integrated machine learning technology to be implemented within Neotree. The Neonatal Encephalopathy Working Group published their study of risk factors for mortality [46], is evaluating the use of the Thompson score and its applicability in the low-resource setting, and is developing a neurodevelopmental follow-up pathway to be implemented in Zimbabwe and Malawi. Ongoing studies from the respiratory distress workshop include an in-depth scoping review of the literature about clinical features and risk factors for neonatal respiratory distress in low-resource settings to be incorporated into the respiratory distress algorithm and evaluation of the uptake of the TRY (T: Tone is good, R: Respiratory distress, and Y: Yes) continuous positive airway pressure algorithm [47] and associated outcomes in Malawi. A similar project is underway for neonatal jaundice. A mixed methods, large-scale evaluation of impact on neonatal quality of care and survival is planned. A key gap within the Neotree system is the potential to link with point-of-care diagnostic technology that is low cost, suitable and adaptable to the low-resource setting; early discussions have been made with potential collaborators.

### Conclusions

This study describes the successful development and implementation of the only known neonatal CDS system incorporated within a bedside data capture system with the ability to deliver up-to-date management guidelines, tailored to local resource availability. The methods used in this study highlighted the importance of collaborative participatory design and South to South learning. Real-world usability testing could further enhance effective implementation. Detailed understanding of the context and setting within which the digital intervention has been implemented is an essential component for sustainability. Effective, motivated local and international partnerships are key to the success of CDS integration into routine practice. Further review of newborn CDS functionality impact and implementation evaluation is planned to guide and inform the development of health system and program strategies to support HCPs, who are overstretched and underresourced, with the ultimate goal of reducing preventable neonatal morbidity and mortality in low-resource settings.

## Appendix

Multimedia Appendix 1 Description of the web editor function of Neotree.

Multimedia Appendix 2 Detailed description of the diagnosis specific algorithms with the associated country-specific management guidelines.

## Abbreviations

CDS: clinical decision support
HCP: health care professional
KCH: Kamuzu Central Hospital
PSSUQ: Post-Study System Usability Questionnaire
SMCH: Sally Mugabe Central Hospital
SUS: System Usability Scale

## Data Availability

Researchers interested in accessing the usability data will first need to send a request to the medical research council of Zimbabwe by email.
