# Supplementary material for: Development and Implementation of Digital Diagnostic Algorithms for Neonatal Units in Zimbabwe and Malawi: Development and Usability Study
Source: JMIR Form Res. 2024 Jan 26;8:e54274. doi: 10.2196/54274 (PMC10858425; doi:10.2196/54274)
Supplement: Multimedia Appendix 1 [file formative_v8i1e54274_app1.docx]

# Supplementary Material 1: Neotree Web editor function

Editor function and the coding:

The Neotree uses an online editor function using “variable expressions” to describe the condition, which is coding depicted by a “$” and brackets (‘’).

For example for Neonatal resuscitation:

- The first question the HCP is asked is if the baby is breathing? – if “no” they are asked to check:
- A = is the airway open?
- B = is the baby breathing?
- C = is the heart rate >100
- D = how is the tone?

The HBB guidelines will surface if any of these are selected:

- Not breathing: $FurtherTriage = 'NotBr'
- Gasping or irregular breathing: $FurtherTriage = 'Gasp'
- Heart rate <100 beats per minute: $FurtherTriage = 'HRLow'
- Floppy: $FurtherTriage = 'Floppy'

*Figure 1* shows the interface of the web editor online and then how this is translated into the management page shown on the Neotree tablet.


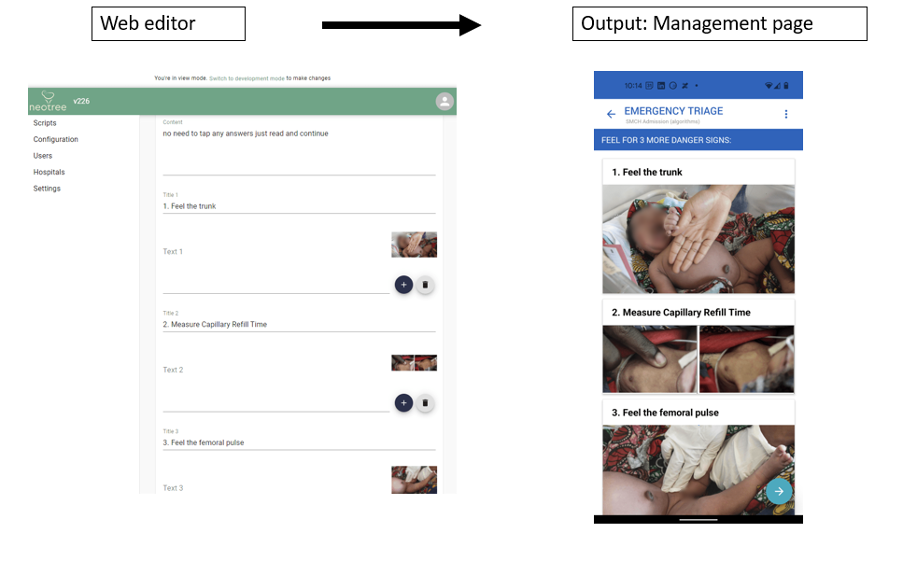


***Figure 1: Web editor interface online***
