# Supplementary material for: Development and Implementation of Digital Diagnostic Algorithms for Neonatal Units in Zimbabwe and Malawi: Development and Usability Study
Source: JMIR Form Res. 2024 Jan 26;8:e54274. doi: 10.2196/54274 (PMC10858425; doi:10.2196/54274)
Supplement: Multimedia Appendix 2 [file formative_v8i1e54274_app2.docx]

# Multimedia Appendix 2: Diagnosis specific algorithms and management guidelines (Malawi and Zimbabwe)

Table of Contents

[Appendix 2: Diagnosis specific algorithms and management guidelines (Malawi and Zimbabwe) 1](#_Toc148973297)

[***1. Thermoregulation: 2***](#_Toc148973298)

[***2. Convulsions 3***](#_Toc148973299)

[***3. Hypoglycaemia 5***](#_Toc148973300)

[***4. Low Birth Weight 7***](#_Toc148973301)

[***5. Prematurity 9***](#_Toc148973302)

[***6. Respiratory distress: 13***](#_Toc148973303)

[***7. Neonatal encephalopathy 19***](#_Toc148973304)

[***8. Suspected Neonatal Sepsis 22***](#_Toc148973305)

[***9. Neonatal Jaundice 27***](#_Toc148973306)

[***10. HIV exposed 31***](#_Toc148973307)

[***11. Congenital abnormalities 36***](#_Toc148973308)

[***References 43***](#_Toc148973309)

Each condition was described during the diagnosis workshop by:

1. *Measurement* (how and when)
2. *Categorisation* (with reference to evidence)
3. *Variable expression* (how the diagnosis is/ will be coded within the Neotree)
4. *Clinical management advice* (with reference to evidence)
5. *Needs for further refinement* (next steps required)

### Thermoregulation:

1. Measured/ recorded: based on temperature recorded on admission
2. Categorisation: according to WHO guidelines [29,30] there are five possible categories for temperature on admission:
   1. Mild hypothermia: temperature 36 - 36.4 degrees
   2. Moderate hypothermia: 32 - 35.9 degrees
   3. Severe hypothermia: < 32 degrees
   4. Normothermia: 36.5 - 37.5 degrees
   5. Hyperthermia: >37.5 degrees
3. Variable Expression:
   1. Algorithm Version2: proposed

Mild Hypothermia: $Temperature > 35.9 and $Temperature < 36.5

Moderate Hypothermia: $Temperature > 31.9 and $Temperature < 36

Severe Hypothermia: $Temperature < 32

1. Management:

Hypothermia Management:

- Warm the baby
  - Skin to skin (Kangaroo Mother Care (KMC) position) or place on warmer or resuscitaire
- Limit heat loss
  - Make sure baby is dry
  - Put on a hat & wrap up the baby
  - If on the resuscitaire put the sides up
  - (consider) The 4 ways by which a baby may lose heat are:
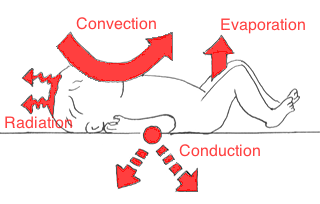


***Figure S1: Heat loss***

- Monitor temperature
  - Check temperature every 15-30 mins or until warm between 36.6-37.5’C
  - Watch out for over-warming (>37.5)
  - Complete the Neotree assessment to assess for signs of infection, apnoea, and hypoglycaemia

Hyperthermia Management:

The baby's temperature is too high! (>37.5)

Do the following & then continue with the Neotree

- Unwrap the baby
- Remove from warmer or skin to skin
- Babies >2500g consider paracetamol or tepid sponging – statement for Malawi only

The neonatal populations are different in the two sites, Malawi admit any baby from home <30 days old. In Zimbabwe it is their first birth admission only.

### Convulsions

1. Measured/recorded: on history and clinical assessment by the healthcare professional (HCP)
2. Categorisation:

If a baby is thought to be having clinical convulsions at any time during the assessment the HCP will state convulsions. Currently there are 4 specific points during the admission process where the HCP can indicate convulsions namely:

- At emergency triage (danger signs)
- Reason for admission
- In the history
- During neurology examination

International and WHO guidance the same [26,31].

1. Variable Expression:

*$DangerSigns = 'Convulsions' or $SymptomReviewNeurology = 'Convulsions' or $Activity = 'Convulsions' or $AdmReason = 'Convulsions'*

1. Management:

| Zimbabwe Management Pages | Malawi Management Pages |
| --- | --- |
| 1. Airway management & oxygen  - Neutral position  - Check for secretions, suction if needed  - Put on oxygen | Refer to management of convulsions flow chart on wall  1. Airway management & oxygen  - Neutral position  - Check for secretions, suction if needed  - Put on oxygen |
| 2. Check blood glucose  - If < 47 mg/dl or < 2.6 mmol/L give IV 10% glucose at 2ml/kg  - If blood glucose monitoring not available give IV 10% glucose at 2ml/kg  - Do full infection screen (blood culture, lumbar puncture, CRP if age is more than 36 hours)  - Take blood for Ca, Mg, U and E’s, FBC | 2. Check blood glucose  - If < 45 mg/dl or < 2.5 mmol/L give IV 10% glucose at 2ml/kg  - If blood glucose monitoring not available give IV 10% glucose at 2ml/kg  - Do full infection screen  - If possible take blood for Ca, Mg, U and E’s, FBC |
| 3. Give anticonvulsants  - Give phenobarbitone 20mg/kg IM  - Repeat loading dose ONCE if necessary  - If seizures ongoing give Phenytoin with a loading dose 20mg/kg  IV  - If seizures still ongoing - give maintenance phenobarbitone 5mg/kg PO/IM  When seizures have stopped and baby stable click the blue arrow to continue | 3. Give anticonvulsants  - Give phenobarbitone 20mg/kg IM  - Repeat loading dose if necessary  - If seizures ongoing give Paraldehyde Dose: 0.2 ml/kg IM, 0.4 ml/kg PR  - If seizures still ongoing - give maintenance phenobarbitone 5mg/kg PO/IM  When seizures have stopped and baby stable click the blue arrow to continue |

Abbreviations:

IV: Intravenous, PO: per oral, IM: Intramuscular, PR: per rectum, Ca: Calcium, Mg: Magnesium, U and E’s: Urea and Electrolytes, FBC: Full blood count, CRP: C-reactive protein

### Hypoglycaemia

1. Measured/recorded: based on information recorded on admission. This is dependent on availability of glucose monitors and testing strips.
2. Categorisation:

This is defined as the baby's blood glucose level being  < 2.5 mmol or 45 mg per deciliter in line with established COIN (Care of the infant and newborn) guidelines [31] and in Zimbabwe they use < 2.6mmol/dL as per EDLIZ (Essential Medicines List and Standard Treatment Guidelines for Zimbabwe) guidelines and WHO [26,32].

- BAPM guidelines [33]: (British Association of Perinatal Medicine)
  - Not tested in first 2-4 hours
  - If <2.5mmol with clinical signs, 2 x <2.0mmol in baby at risk of hypoglycaemia and no clinical signs, <1.0mmol at any time - treat
- ETAT+ guidelines:  if can breast feed and <2.2mmol give feed, if <1.1 treat with IV glucose, if 1.1-2.2 consider immediate nasogastric tube (NGT) insertion and feed with expressed breast milk (EBM)

Categorised currently according to above guidelines as follows

- **Hypoglycaemia** blood sugar less than 2.5mml or 45 mg for Malawi and <2.6 mmol in Zimbabwe.
  - **Hypoglycaemia Symptomatic**
  - **Hypoglycaemia NOT symptomatic**

1. Variable Expression:

- At risk of hypoglycemia: $BirthWeight < 2500 or $Gestation < 37 or $PregConditions = ‘DM’ or $ANSteroids = ‘Y’ or $AdmReason = ‘NE’
- Hypoglycemia symptomatic: ($BloodSugarmmol < 2.6 or $BloodSugarmg < 47) and $HypoSymptoms = true
- Hypoglycemia asymptomatic: ($BloodSugarmmol < 2.6 or $BloodSugarmg < 47) and $HypoSymptoms = false

1. Management:

**HYPOGLYCAEMIA NOT SYMPTOMATIC**

| Zimbabwe Management Pages | Malawi Management Pages |
| --- | --- |
| 1. Continue Breast Feeding or giving EBM by cup  - Monitor blood glucose 30 mins after feed | 1. Continue Breast Feeding or giving EBM by cup  - Monitor blood glucose 30 mins after feed |
| 2. Increase frequency of feeds e.g. from 3 to 2 hourly or from 2 to 1 hourly | 2. Increase frequency of feeds e.g. from 3 to 2 hourly or from 2 to 1 hourly |
| 3. Monitor blood glucose before next feed or immediately if any symptoms!  - If 3 normal blood sugars (2.6 - 8.3mmol), monitoring can be stopped | 3. Monitor blood glucose before next feed or immediately if any symptoms!  For further management refer to the wall chart |

**HYPOGLYCAEMIA - (SYMPTOMATIC)**

| Zimbabwe Management Pages | Malawi Management Pages |
| --- | --- |
| 1. Give dextrose bolus  - 2ml/kg of 10% dextrose IV over 5 minutes  - If no IV-line feed baby via oral gastric tube (OGT) or nasogastric tube (NGT) as per feed chart | Do the following and refer to page 311 COIN, Chart C  1. Give dextrose bolus  - 2ml/kg of 10% dextrose IV over 5 minutes  - If no IV line, give the same IV dextrose by gastric tube. |
| 2. Give Maintenance fluids  For fluid and rate see Wall Chart | 2. Give Maintenance fluids  For fluid and rate see Wall Chart |
| 3. Monitor Blood Glucose after 30 minutes | 3. Monitor Blood Glucose after 30 minutes  For further management refer to the wall chart |


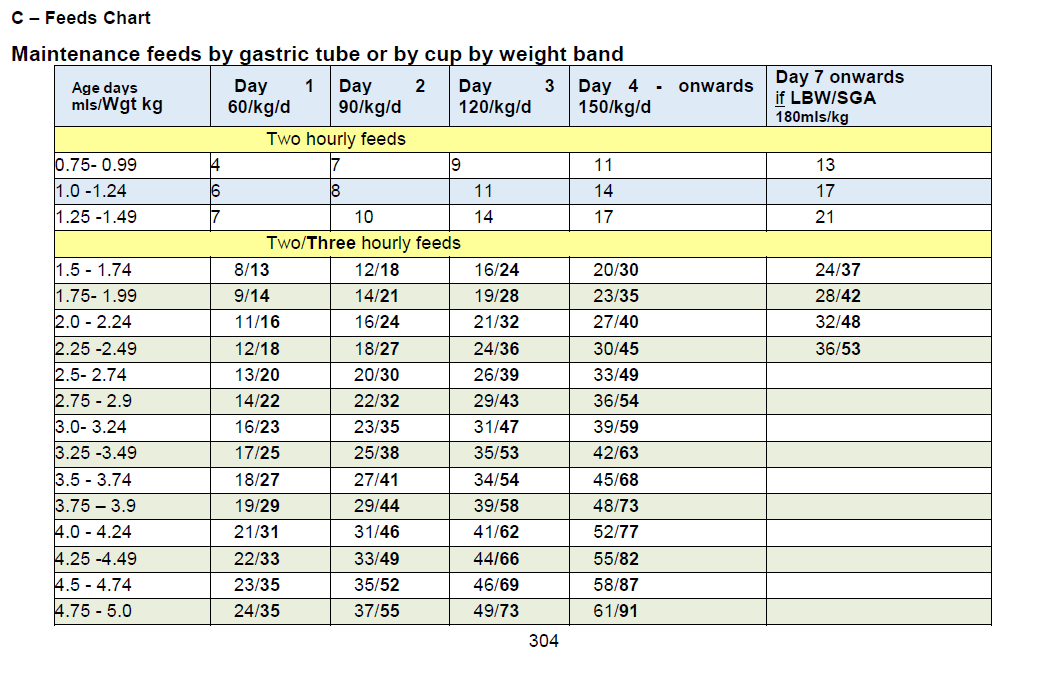

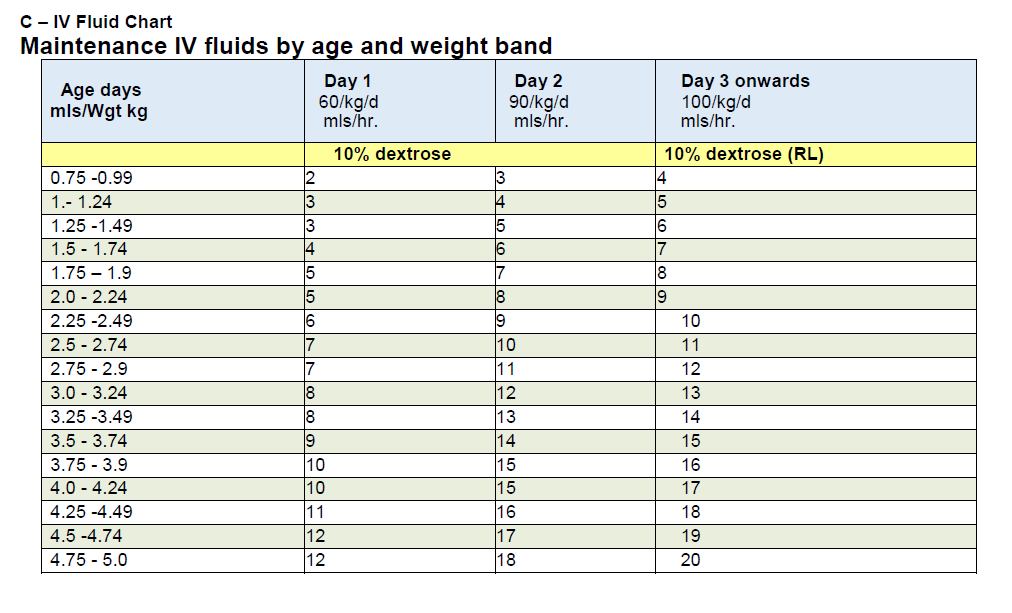


***Figure S2: Feed and fluid chart examples***

### Low Birth Weight

1. Measured/recorded:

- Inborn babies: on admission (grammes) by HCP using available scales
- Outborn babies: as recorded by the referring hospital (g)

1. Categorisation:

According to WHO/international guidelines (ICD-10) [34] into 5 groups.

1. Low birth weight (LBW) defined as birth weight greater than 1499g and less than 2500g
2. Very low birth weight (VLBW) defined as birth weight greater than 999g and less than 1500g
3. Extremely low birth weight (ELBW) defined as birth weight less than 1000g
4. Appropriate weight for gestational age
5. Big baby defined as birth weight greater than 4000g
6. Variable Expression:

- ***LBW*** = $BirthWeight > 1499 and $BirthWeight < 2500
- ***VLBW*** = $BirthWeight > 999 and $BirthWeight < 1500
- ***ELBW*** = $BirthWeight < 1000
- ***HBW*** = $BirthWeight > 4000

HCP chosen diagnosis: $Diagnoses = ‘LBW’ (this is assumed to mean any baby with a weight less than 2500g)

1. Management:

| Zimbabwe Management Pages | Malawi Management Pages |
| --- | --- |
| Manage this baby according to the weight as below:  ≥ 1800g:  - May require cup feeds  - Skin-skin & kangaroo care for thermoregulation  - If the baby is feeding well with assistance, warm and not exhibiting any danger signs, the baby can stay with their mother  - If the baby is not feeding well by breast, bottle, or cup or exhibiting any danger signs the baby should stay in neonatal unit  Less than 1800g:  - Needs admission to neonatal unit for close observation  - 1500-1799g if not feeding well by breast start cup feeds (change from 2000)  - Those <1500g should be fed by NGT and feeds advanced slowly as per the table | Manage this baby according to the weight as below:  1800-2500g:  - These babies are usually strong enough to feed normally on the breast after birth  1500-1799g  - May require cup feeds  - Skin-skin & kangaroo care for thermoregulation  - If the baby is feeding well with assistance, warm and not exhibiting any danger signs, for ambulatory KMC  - If the baby is not feeding well by breast, bottle, or cup or exhibiting any danger signs the baby should stay in special care nursery  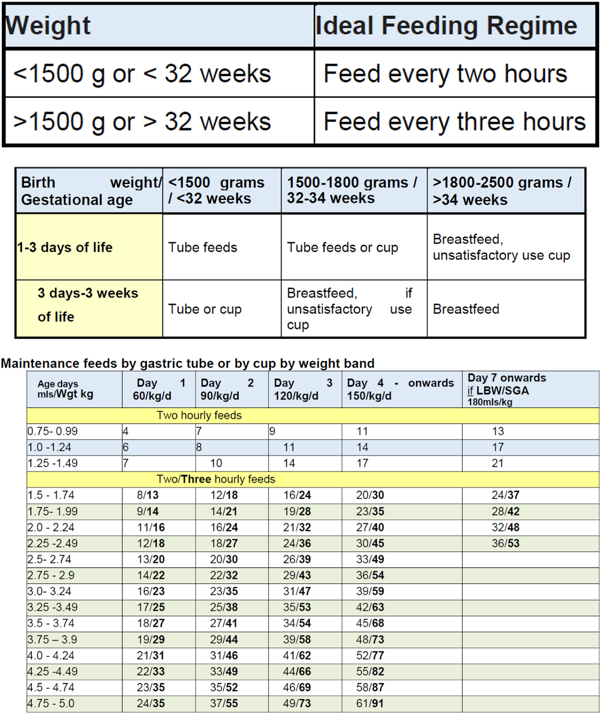  Less than 1500g  - Needs admission to special care nursery for close observation |

### Prematurity

1. Measured/recorded: from history based most commonly on last menstrual period, and where available antenatal dating scans and clinical examination after birth
2. Categorisation:

According to WHO/international guidelines (ICD-10) [34] into 5 groups

1. Premature ≤37 weeks
2. Very premature ≤32 weeks
3. Extremely premature ≤28 weeks
4. Term
5. Postdates
6. Variable Expression:

- ***Prematurity*** (32-36 weeks):
  - $Gestation > 31.9 and $Gestation < 36.1
- ***Very Premature*** (28-31 weeks):
  - $Gestation > 27.9 and $Gestation < 31.1
- ***Extremely Premature*** (<28 weeks)
  - $Gestation < 28

*HCP chosen diagnosis* $Diagnoses = 'PREM'

1. Management:

| Zimbabwe Management Pages | Malawi Management Pages |
| --- | --- |
| Most stable larger preterm babies (>1500g) not needing oxygen may safely be nursed with their mother in Kangaroo Care.  Remember to perform Ballard Score- images are for neuromuscular followed by physical maturity assessment  - (Ballad score images in script)  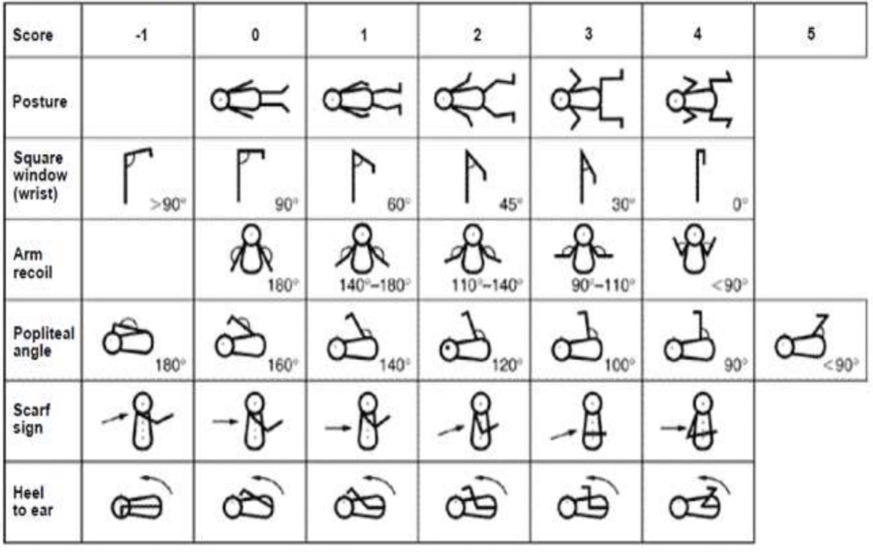 | 1. Thermoregulation  If needing oxygen  - for babies < 1800 g use radiant warmer + intermittent KMC  - for babies > 1800 g radiant warmer  If not needing oxygen  - for babies < 1800g KMC  - for babies 1800 - 2500 ambulatory KMC  Remember to perform Ballard Score- images are for neuromuscular followed by physical maturity assessment  - (Ballad score images in script)    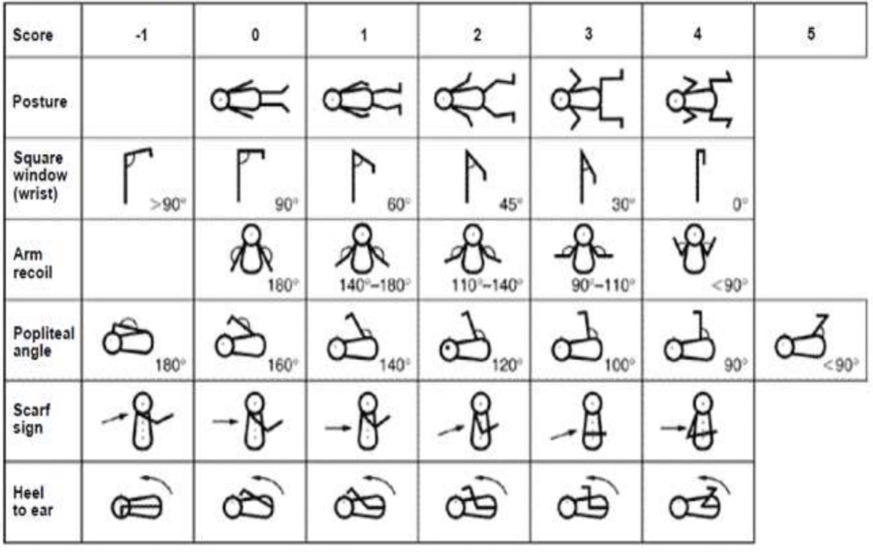 |
| WARM CHAIN is maintained:  • Keep the radiant warmer or resucitaire on  • For babies < 32 weeks deliver into plastic bag before drying if available. If not, dry infant in warm  towel and wrap in another dry warm towel.  • Put skin to skin with mother and cover both with blanket  • Keep room/transport incubator/ambulance (if transporting) as warm as possible.  • Ensure baby has a hat & socks, and perform procedures in the incubator.  1. Thermoregulation  Keep the baby warm aiming between 36.5-37.5’C by Skin to skin (KMC position) or place on radiant warmer or incubator if available  Mild hypothermia: 36-36.4 --> cause for concern,  warm the baby  Moderate hypothermia: 32-35.9--> DANGER  • rewarm baby,  • check blood glucose  Severe hypothermia <32, outcome likely to be poor:  • Rewarm baby,  • Check blood glucose  2. Respiratory support  • Oxygen / CPAP  • CPAP >28 weeks or > 1000g and passes the TRY CPAP algorithm (image in script)  • Remember preterms may have slightly reduced tone due to their prematurity  • Early CPAP for babies between 28 - 32 weeks  • Prevention of apnoea:   - Give Caffeine Citrate 20mg/kg orally or slowly by intravenous route over 30 minutes and a maintenance dose of 5mg/kg/day to all preterm babies<32 weeks. Give until 34 weeks postnestrual age. - If Caffeine citrate is not available give a loading dose of aminophylline at 6mg/kg over 20 minutes followed by a maintenance dose of 2.5mg/kg every 12 hours | 2. Respiratory support  Oxygen / CPAP  CPAP >28 weeks or > 1000g and passes the TRY CPAP algorithm (image in script)  Remember preterms may have slightly reduced tone due to their prematurity  Early CPAP for babies between 28 - 32 weeks  Give aminophylline (or caffeine if available) to prevent apnoeas  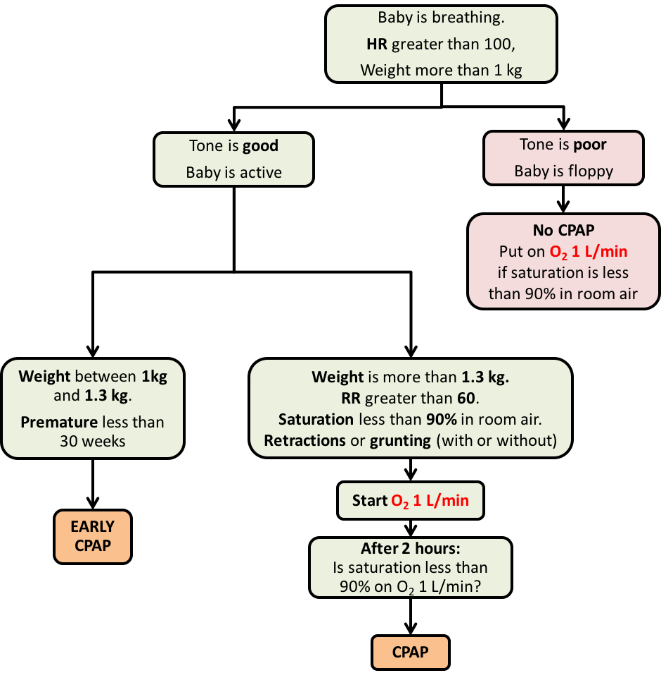 |
| 3. Feeding support  • If able to suck GIVE breast feed  • If not able to suck give cup feeds or consider OGT  • OGT for all babies < 1500g, RR > 60 and needing respiratory support  • OGT if on CPAP  • Consider IV fluids in smaller babies breathing > 80 bpm | 3. Feeding support  If able to suck can breast feed  If not able to suck give cup feeds or consider OGT  OGT for all babies < 1500g, RR > 60 and needing respiratory support  OGT if on CPAP  Consider IV fluids in smaller babies breathing > 80 bpm  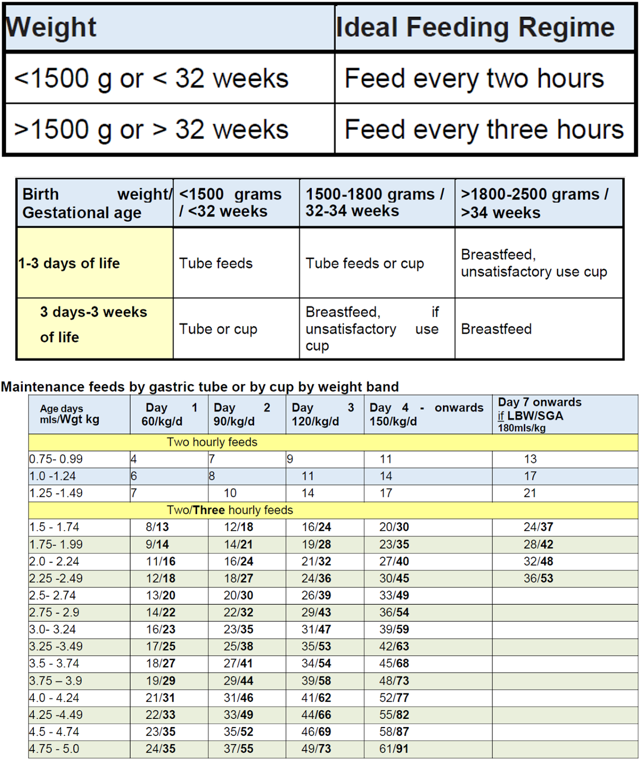 |

### Respiratory distress

1. Measured/recorded: based on information recorded on admission
2. Categorisation:

Algorithm Version1 (*Phase 1 development*) for respiratory distress was as follows. This algorithm was developed using a combination of guidelines (COIN guidelines (2017)) and clinical knowledge (CC).

Six possible diagnoses relating to respiratory distress namely:

1. Respiratory distress syndrome (RDS)
   - Term with respiratory distress (RD) or preterm with RD
2. Possible meconium aspiration
3. Transient tachypnoea of the newborn (TTN)
4. Congenital pneumonia (CPN)
5. Pneumonia/Bronchiolitis
6. Pneumonia/Bronchiolitis/PJP


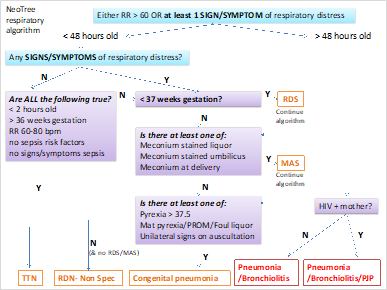


Following the Delphi study, it was concluded that trying to define the specific respiratory diagnosis is too challenging in LRS’s and this should be defined as generic “Respiratory distress” and determined by gestation; Term with Respiratory distress and Preterm with Respiratory distress [23].

**Phase 2 development SME workshops concluded:**

**Respiratory distress of the newborn (Term or Preterm)**

1. *Measured/defined:*

Babies who had a respiratory rate greater than 60 breaths per minute OR at least one other sign or symptom of respiratory distress (below) were defined as having respiratory distress defined as term, if ≥37 weeks, and preterm if under 37 weeks gestation.

Signs/ symptoms of respiratory distress included:

Emergency Triage:

- Grunting
- Cyanosis
- Respiratory rate over 60
- Saturations less than 90% in air
- Saturations less than 90% in oxygen

Signs of Respiratory distress on examination:

- Nasal Flaring ‘NFL’
- Grunting ‘GR’
- Head nodding ‘HN’
- Tracheal tug ‘TT’
- Gasping ‘Gasp’

- Chest indrawing or chest recessions ‘CHI’

Symptom review:

- History of fast/ laboured breathing ‘DIB’
- History of noisy breathing ‘NBr’
- Apnoea ‘Apn’
- Blue episodes ‘BE’
- Cough ‘Cough’

1. Variable Expression:

- Term with respiratory distress (RD)
  - $Gestation >= 37 and ($RR > 60 or $DangerSigns = 'Grun' or $DangerSigns = 'Cyan' or $SatsAIr < 90 or $SatsO2 < 90 or $RespSR = 'DIB' or $RespSR = 'BE' or $RespSR = 'Apn' or $RespSR = 'Cough' or $RespSR = 'NBr' or $SignsRD = 'TT' or $SignsRD = 'NFL' or $SignsRD = 'CHI' or $SignsRD = 'HN' or $SignsRD = 'GR' or $SignsRD = 'Gasp')

- Preterm with RD
  - $Gestation < 37 and ($RR > 60 or $DangerSigns = 'Grun' or $DangerSigns = 'Cyan' or $SatsAIr < 90 or $SatsO2 < 90 or $RespSR = 'DIB' or $RespSR = 'BE' or $RespSR = 'Apn' or $RespSR = 'Cough' or $RespSR = 'NBr' or $SignsRD = 'TT' or $SignsRD = 'NFL' or $SignsRD = 'CHI' or $SignsRD = 'HN' or $SignsRD = 'GR' or $SignsRD = 'Gasp')

1. Management:


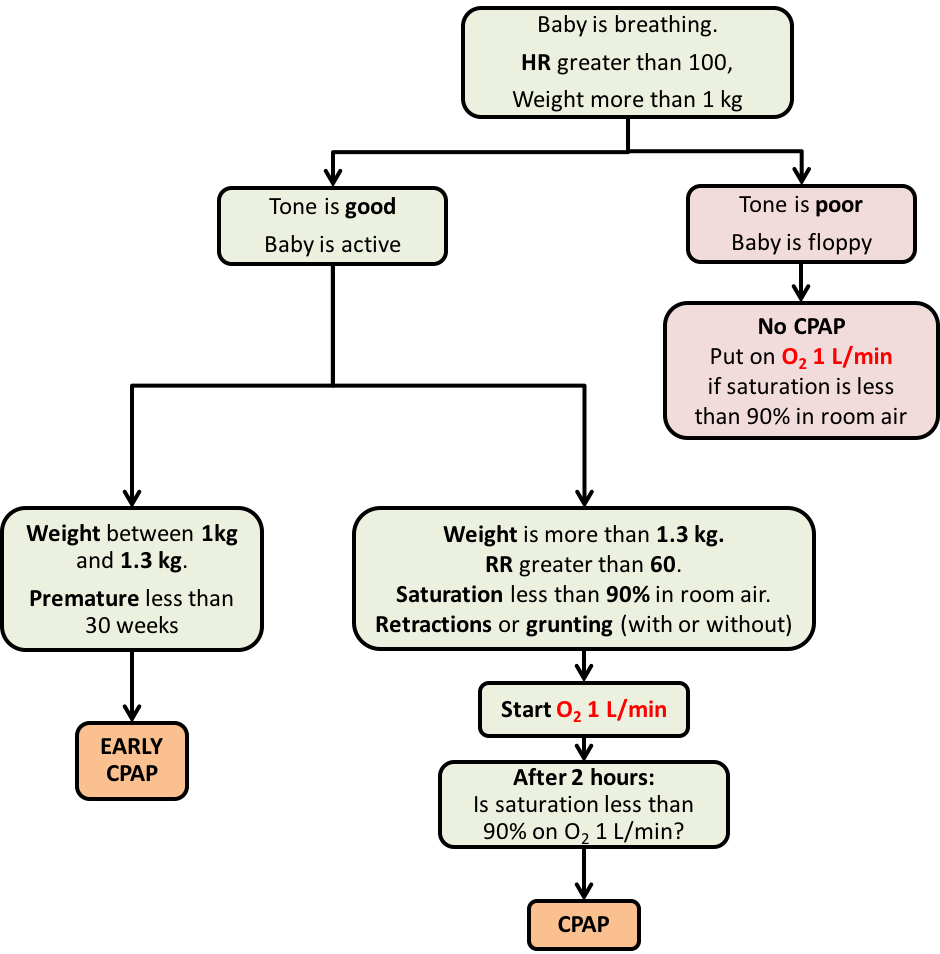


***Figure S3: TRY CPAP algorithm***

**Zimbabwe**

| Preterm with RD | Term with RD |
| --- | --- |
| Care must be given to thermoregulation as per preterm guidelines  Remember to perform Ballard Score- images are first Neuromuscular followed by Physical maturity assessment  1. Airway and Respiratory support  Position the airway in neutral  If saturations < 90% put on oxygen  If distress is significant & > 1kg consider CPAP according to the TRY CPAP algorithm.  Start on Caffeine if baby is <34 weeks old or <1500g  Consider surfactant if >1000g with severe respiratory distress requiring CPAP and an oxygen requirement FiO2>35% | 1. Airway and Breathing  Position airway in neutral  Apply nasal prong oxygen to maintain saturations 90-95%  Consider CPAP as per TRY CPAP algorithm  if > 1kg and good tone and if the RR>60, Room oxygen saturations are < 90% or if there are retractions and grunting per flowchart below. |
| 2. Feeding support  If breathing 60-80 bpm use cup/OGT  If needing CPAP use OGT  If breathing > 80 bpm consider IV fluids | 2. Feeding support:  If >34 weeks or >1500g and NOT on CPAP use cup/OGT feeding until RR <60 bpm  OGT for all babies < 1500g, RR > 60 and needing respiratory support  OGT if on CPAP  Consider IV fluids if respiratory rate > 80 bpm |
| 3. Get IV access and start antibiotics  Antibiotics are recommended in all preterms with RDS if mother confirmed sepsis or suspected chorioamnionitis OR spontaneous preterm labour OR with 2 or more of the following risk factors for sepsis  Risk factors:  Rupture of membranes >18hrs  Group B Streptococcus (GBS) sepsis in previous baby or documented GBS carriage in this pregnancy (urine or vaginal swab)  Born before arrival  Meconium stained liquor  Take blood culture  Give x pen (X penicillin) 50000 units twice daily (BD) and gentamicin 4mg/kg once daily (OD) (if <2000g) | 3. Antibiotics:  - If respiratory distress started greater than 4 hours old (if there are no other conditions requiring antibiotics earlier than 4 hours old) and ongoing respiratory distress:  Take blood culture before starting antibiotics  Obtain IV access & commence  X pen 50000 units BD and  Gentamicin 3mg/kg OD (if <2500g) OR Gentamicin 5mg/kg OD (if >2500g)  4. Meconium  Most meconium aspiration occurs in utero before the baby is born, so more likely if there was foetal distress  Suspect meconium aspiration IF sick with significant respiratory distress +/- signs of birth asphyxia and consider starting this baby on antibiotics  Floppy babies with severe hypoxic ischemic encephalopathy (HIE) and Meconium aspiration are unlikely to benefit from CPAP |

**Malawi**

| Preterm with RD | Term with RD |
| --- | --- |
| Care must be given to thermoregulation as per preterm guidelines  Remember to perform Ballard Score- images are first Neuromuscular followed by Physical maturity assessment  1. Airway and Respiratory support  Position the airway in neutral  If saturations < 90% put on oxygen  If distress is significant & > 1kg consider CPAP according to the TRY CPAP algorithm.  Start on Aminophylline if baby is < 34 week or <1.5kg  Consider surfactant if >1000g with severe respiratory distress requiring CPAP and an oxygen requirement >35% | 1. Airway and Breathing  Position airway in neutral  Apply nasal prong oxygen to maintain saturations 90-95%  Consider CPAP as per TRY CPAP algorithm  if > 1kg and good tone but if the RR>60, Room oxygen saturations are < 90% or if there are retractions and grunting per flowchart below. |
| 2. Feeding support  If breathing 60-80 bpm use cup/OGT  If needing CPAP use OGT  If breathing > 80 bpm consider IV fluids | 2. Feeding support:  If >34 weeks or >1500g and NOT on CPAP use cup/OGT feeding until RR <60 bpm  OGT for all babies < 1500g, RR > 60 and needing respiratory support  OGT if on CPAP  Consider IV fluids if respiratory rate > 80 bpm |
| 3. Get IV access and start antibiotics  Antibiotics are recommended in all preterms with RDS if mother confirmed sepsis or suspected chorioamnionitis OR spontaneous preterm labour OR with 2 or more of the following risk factors for sepsis  Risk factors:  Take blood culture (if possible) before starting antibiotics  Obtain IV access & commence  X pen 50000 units iv BD and  Gentamicin 4mg/kg OD (if <2000g) OR Gentamicin 5mg/kg OD (if >2kg)  For senior doctor review for duration of antibiotics | 3. Antibiotics:  - If respiratory distress started greater than 4 hours old (if there are no other conditions requiring antibiotics earlier than 4 hours old) and ongoing respiratory distress:  Take blood culture before starting antibiotics  Obtain IV access & commence  X pen 50000 units BD and  Gentamicin 4mg/kg OD (if <2000g) OR Gentamicin 5mg/kg OD (if >2kg)  4. Meconium  Most meconium aspiration occurs in utero before the baby is born, so more likely if there was foetal distress  Suspect meconium aspiration IF sick with significant respiratory distress +/- signs of birth asphyxia and consider starting this baby on antibiotics  Floppy babies with severe HIE and Meconium aspiration are unlikely to benefit from CPAP |

E: Ongoing work

Includes an in-depth scoping review of the literature on clinical features and risk factors for neonatal respiratory distress in low resource settings to be incorporated into the respiratory distress algorithm and evaluation of uptake of the TRY-CPAP algorithm.

### Neonatal encephalopathy

**Hypoxic Ischaemic Encephalopathy (Zimbabwe) or Neonatal Encephalopathy (Malawi)**

1. Measured/recorded: based on information recorded on admission
2. Categorisation:

This is based on the Thompson score, with triggers for conducting the Thompson score based on Mugwagwa *et al’s* (manuscript in progress) work which was also informed by Evans et al 2020.

*Triggers for conducting Thompson score:*

In Zimbabwe:

Gestational age >= 37 weeks AND (Apgar score < 7 at 5 minutes OR neonatal heart rate at admission less than 100b/m OR duration of resuscitation greater than 10 mins OR neonatal encephalopathy/HIE as admission reason OR born outside the hospital

In Malawi:

Gestational age >= 37 weeks AND (Apgar score < 7 at 5 minutes OR neonatal heart rate at admission less than 100b/m OR duration of resuscitation greater than 10 mins OR neonatal encephalopathy as admission reason

1. Variable Expression:

Thompson Score ≥ 11 triggers “HIE” as diagnosis

Thompson Score > 0 and < 11, then “Suspected HIE” is triggered which has the same management as HIE.

Risk factors

- Birth Resuscitation: BVM >5mins / CPR>10 mins -($Resus = 'BVM' and $LengthResus > 5) or $Resus = 'CPR' or $LengthResus > 10
- Foetal distress $Reason = 'FD'
- Apgar at 5 mins<7 (as per COIN) $Apgar5 < 7
- Convulsions $DangerSigns = 'Conv' or $SRNeuroOther = 'Conv' or $Activity = 'Conv'
- Coma  $Activity = 'Coma'
- Lethargy  $Activity = 'Leth'
- Hypotonia and gestation >34/40 weeks - $Gestation > 33 and $Tone = 'Low'

1. Management:

*
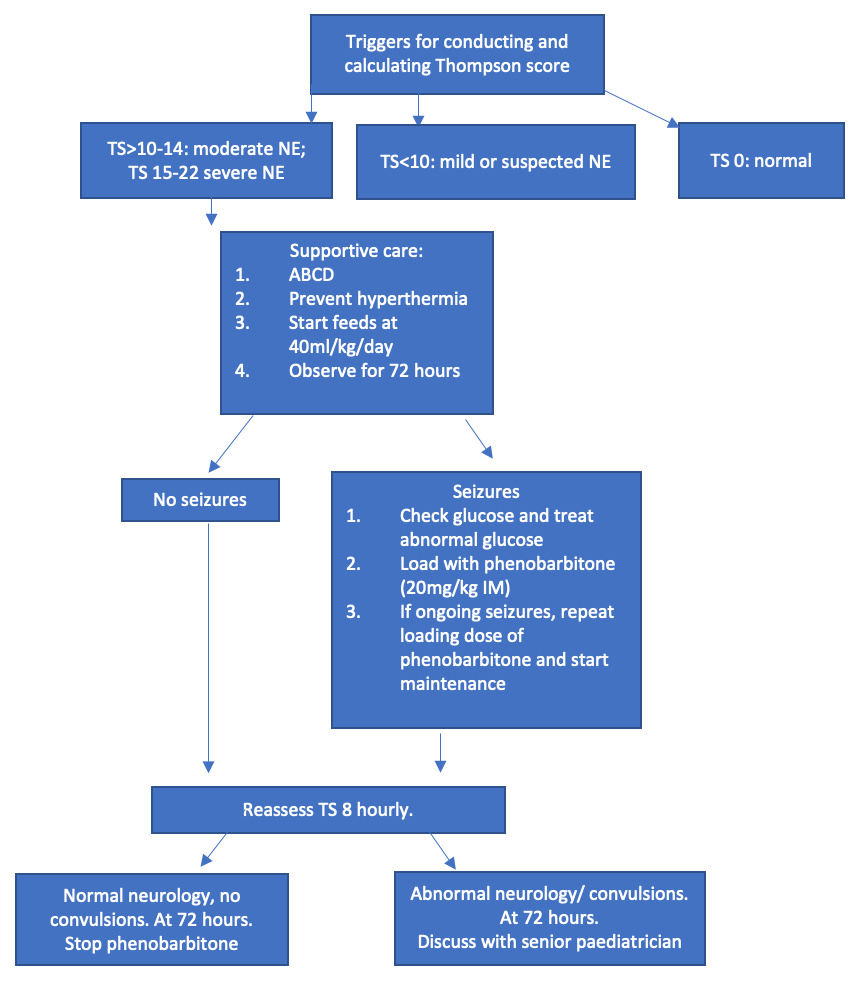
*

| Zimbabwe Advice | Malawi Advice |
| --- | --- |
| 1. Supportive care:  ABCD (Airway, Breathing, Circulation, "Don't forget Glucose")  Prevent hyperthermia  Start feeds to maximum of 40ml/kg/day  Observe for 72 hours | 1. Supportive care:  ABCD  Prevent hyperthermia  Start feeds to MAXIMUM of 40ml/kg/day  Observe for 72 hours |
| 2. If the baby has seizures  Check glucose if < 47 mg/dl or < 2.6 mmol/L give IV 10% glucose at 2ml/kg  Load with phenobarbitone (20mg/kg IM)  Check U&Es,Ca,Mg,PO4  If ongoing seizures, repeat loading dose of phenobarbitone and start maintenance (5mg/kg oral or IM)  Monitor respirations | 2. If the baby has seizures  Check glucose and treat abnormal glucose  Load with phenobarbitone (20mg/kg IM)  If ongoing seizures, repeat loading dose of phenobarbitone and start maintenance |
| 3. Reassess Thompson score 8 hourly.  Start antibiotics. Covering for meningitis  If baby has normal neurological examination after 72 hours and has been seizure free for 48 hours, stop phenobarbitone.  If baby has abnormal neurology/ convulsions at 72 hours to discuss with senior paediatrican | 3. Reassess TS 8 hourly.  Normal neurology, no convulsions at 72 hours ->Stop phenobarbitone  If abnormal neurology/ convulsions at 72 hours -> Discuss with senior paediatrician |

E: Ongoing work is planned in the Neonatal Encephalopathy Working Group including refinement of the algorithm and developing a neurodevelopmental follow-up pathway to be implemented in Zimbabwe and Malawi.

### Suspected Neonatal Sepsis

1. Measured/recorded: according to history and clinical examination
2. Categorisation and
3. Variable Expression:

Zimbabwe uses the EDLIZ table to assist healthcare workers in determining whether they should expect neonatal sepsis. There are major criteria and minor criteria which add up to trigger the algorithm. If the healthcare worker selects at least 1 major criteria or 2 minor criteria, the “suspected neonatal sepsis” diagnosis algorithm will trigger.

Major criteria:

1. Confirmed sepsis or chorioamnionitis in mother
2. Confirmed or suspected sepsis in twin
3. Seizures
4. Severe Respiratory Distress in a term infant
5. Signs of shock

Minor criteria:

1. ROM >18h
2. Spontaneous Preterm Birth
3. GBS sepsis in previous baby or documented GBS carriage in this pregnancy (urine or vaginal swab)
4. Born Before Arrival
5. Meconium-Stained Liquor
6. Respiratory distress that is not obviously related to: 1. environmental hypothermia 2. “delayed transition to extra-uterine life” i.e., mild to moderate respiratory distress apparent soon after birth that is improving with time.
7. Hypoxia
8. Apnoea
9. Hypoglycaemia/Hyperglycaemia not otherwise explained
10. Temperature instability not explained by environmental factors
11. Acidosis not obviously related to HIE
12. Unexplained bleeding or thrombocytopenia
13. Mild encephalopathy/Altered responsiveness
14. Altered tone not otherwise explained
15. Feed intolerance/feeding difficulty
16. Abnormal heart rate (<90 or >160)
17. Jaundice in first 24h

The format of the EDLIZ table in Zimbabwe was highly acceptable in user testing at SMCH and was recommended to use a similar format in Malawi, however, by implementing Malawi-specific (COIN) guidelines for Neonatal Sepsis diagnosis. Instead of major and minor criteria however, it is broken down into the following categories:

1. Antenatal risk factors
   1. Fever >38 in mother
   2. Rupture of membranes >18h
   3. Offensive Liquor
2. Signs and symptoms of sepsis
   1. Temperature <35.5 or >37.5
   2. Bulging Fontanelle
   3. Grunting
   4. Nasal Flaring
   5. Crepitations in the lungs
   6. Fast breathing
   7. Chest indrawing
   8. Umbilical redness extending to the periumbilical skin or umbilicus draining pus
   9. Reduced movement of limbs
   10. Many skin pustules/big boil (abscess)
3. Signs and symptoms of meningitis
   1. Drowsiness
   2. Lethargy/unconscious
   3. Persistent irritability
   4. High pitched cry
   5. Convulsions
4. Shock (danger signs)
   1. Cold hands and feet
   2. Capillary refill time > 3 seconds
   3. Fast and weak pulse

If the clinician using the app clicks on any of the signs/risk factors, the algorithm will trigger.

1. Management:

***The suspected neonatal sepsis management is slightly different for Zimbabwe and Malawi. First, we have Zimbabwe:***

1.Perform blood cultures PRIOR to giving antibiotics if possible

- REMEMBER TO WRITE NEOTREE NUMBER ON BLOOD CULTURE BOTTLE

2. Antibiotics

- All babies with suspected sepsis should receive intravenous antibiotics. If antibiotics are started in the first 48 h use:
- X-Pen/Benzyl Penicillin and Gentamicin. See full guideline below for dosing and duration
- Even if there are no risk factors for sepsis on admission, babies should be reassessed for signs of sepsis daily during admission

3. Supportive care

- Check blood sugar and provide feeding support if needed
- Give 2.5 mL/kg 10% dextrose bolus if blood glucose <2.6 and baby symptomatic
- Give 10mL/kg fluid bolus if shocked
- Thermoregulation: aim for body temperature between 36.5 - 37.5
- If baby has fever (T >38.5) remove from warmer and unwrap
- Provide respiratory support as needed

Full guideline for reference:

1. Perform cultures PRIOR to giving antibiotics if possible

- REMEMBER TO WRITE NEOTREE NUMBER ON BLOOD CULTURE BOTTLE
- Follow the unit blood culture taking guidelines, with special care to minimise contamination and ensure an adequate blood volume in the bottle (1-2 ml). If it is impossible to perform the culture, antibiotics should not be delayed, but this is sub-optimal medicine.
- If the suspicion of meningitis is strong, one should perform a lumbar puncture before starting antibiotics if possible.

2. Antibiotics

- If antibiotics are started in the first 48 h use: X-Pen/Benzyl Penicillin and Gentamicin
- Gentamicin dosing (Give as slow IV or infusion)
  - <2kg Birth weight : use 5 mg kg/dose
    - <1 kg : 48 hourly dosing for first two weeks, then 36h dosing
    - 1-2 kg <1 week old 48h dosing, >1-week 36h
  - >2kg birth weight : use 4mg/kg dose
    - <1 week : 24h dosing
    - >1 week 24h dosing-48h dosing
- NB: increase dose interval by 12 h if at risk of Acute Kidney Injury (e.g., HIE or patient on other nephrotoxic drugs).
- One should perform a gentamicin trough level after every third dose. Monitor urine output and renal function in babies who remain on aminoglycosides.
- Benzyl Penicillin dosing : 50-100 000 IU/kg/dose slow IV ( use higher dose in meningitis or GBS)
  - Frequency:
    - - Preterm : 1st week 12h then 1-3 weeks 8h
      - Term : 12 hourly for first week
  - (NB Ampicillin at 50-100mg/kg is an alternative to benzyl Penicillin with the same dosing intervals, use higher dose in meningitis or GBS)
- (If there are concerns of cellulitis/arthritis or osteomyelitis rather use cloxacillin and gentamicin: stop gentamicin if blood culture excludes Gram negative organism)
  - Cloxacillin dose 25-50 mg/kg (use 100mg/kg if osteitis or intracranial infection)
  - 1st week of life : 12h
  - 2nd to 4th  week of life : 8h

Duration of antibiotics:

- Most babies who have empirical antibiotics started for suspected sepsis do not in fact have an infection
- Stop antibiotics at 36-48h if they fulfil all these criteria:
  1. No growth has been obtained on the blood culture so far
  2. The CRP level done at 36-48 h is normal (if available)
  3. The baby does not have clinical signs of sepsis
- Babies with a positive blood culture should have a lumbar puncture (LP) done.
- If there is no meningitis antibiotics should be continued for at least 7-10 days, and only terminated when the child is well. If CRP is being monitored ensure it has normalised before stopping antibiotics.
- If the child has Gram-positive meningitis (including GBS or Staphylococcus aureus) or Staphylococcus aureus septicaemia continue antibiotics for at least 14 days
- If Gram negative meningitis (E. coli or Klebsiella);  continue antibiotics for at least 21 days

3. Supportive care

- Check blood sugar and provide feeding support if needed
- Give 2.5 mL/kg 10% dextrose bolus if blood glucose <2.6 and baby symptomatic
- Give 10mL/kg fluid bolus if shocked (Normal saline or Ringer’s lactate)
- Thermoregulation: aim for body temperature between 36.5 - 37.5
- If baby has fever (T >= 38.5) remove from warmer and unwrap

***For Malawi the management for Neonatal Sepsis (here there is no “suspected”) is as follows:***

1. Perform cultures BEFORE giving antibiotics if possible

- Follow the unit blood culture taking guidelines, with special care to minimise contamination and ensure an adequate blood volume in the bottle (1-2 ml).
- If it is impossible to perform the culture, antibiotics should not be delayed, but this is sub-optimal medicine.
- If the suspicion of meningitis is strong, one should perform a lumbar puncture before starting antibiotics if possible.

2. Antibiotics

Start antibiotics:

- X-Penicillin 50,000 iu/kg 12 hourly IV/IM and

- Gentamicin 3mg/kg OD (if <2500g) OR Gentamicin 5mg/kg OD (if >2500kg)

- Change frequency of Penicillin to 6 hourly if > 7days age
- Change dose of gentamicin to 7.5mg/kg every 24 hours if > 7 days age

Length of antibiotics:

- Stop at five days if baby well and all investigations are negative
- If blood culture comes back negative before 5 days and baby is well then stop antibiotics when negative culture result received.
- If clinically unwell, give 7-10 days, though meningitis or suspected bone/joint infections require longer
- Give 14-21 days with high suspicion of meningitis or positive CSF culture result
- If GBS positive cerebral spinal fluid (CSF) result treat for 14 days
- If E.coli or Klebsiella give 21 days

3. Supportive care

- Check blood sugar and provide feeding support if needed
- Give 2.5 mL/kg 10% dextrose bolus if blood glucose <2.6 and baby symptomatic
- Give 10mL/kg fluid bolus if shocked
- Thermoregulation: aim for body temperature between 36.5 - 37.5
- If baby has fever (T >= 38.5) remove from warmer and unwrap
- Provide respiratory support as needed

E: Ongoing work

The Neonatal Sepsis Working Group is currently in the process of an in-depth review of neonatal sepsis guideline use within low-resource settings, a scoping review and developing a robust clinical prediction model with integrated machine learning technology to be implemented within the Neotree.

### Neonatal Jaundice

1. Measured/recorded: A baby is defined as having clinical jaundice if the HCP states the baby’s skin colour is yellow on clinical examination.
2. Categorisation:

Pathological jaundice:

- Day 1 of life (<24 hours of age)
- Deep jaundice (yellow palms and soles)
- Prematurity (<35 weeks or < 2.5 kg)

Physiological jaundice:

- The baby has physiological jaundice which is characterised by yellow eyes and skin and no signs of pathological jaundice.

Prolonged jaundice

- Prolonged jaundice > 14 days term or >21 days preterm infants


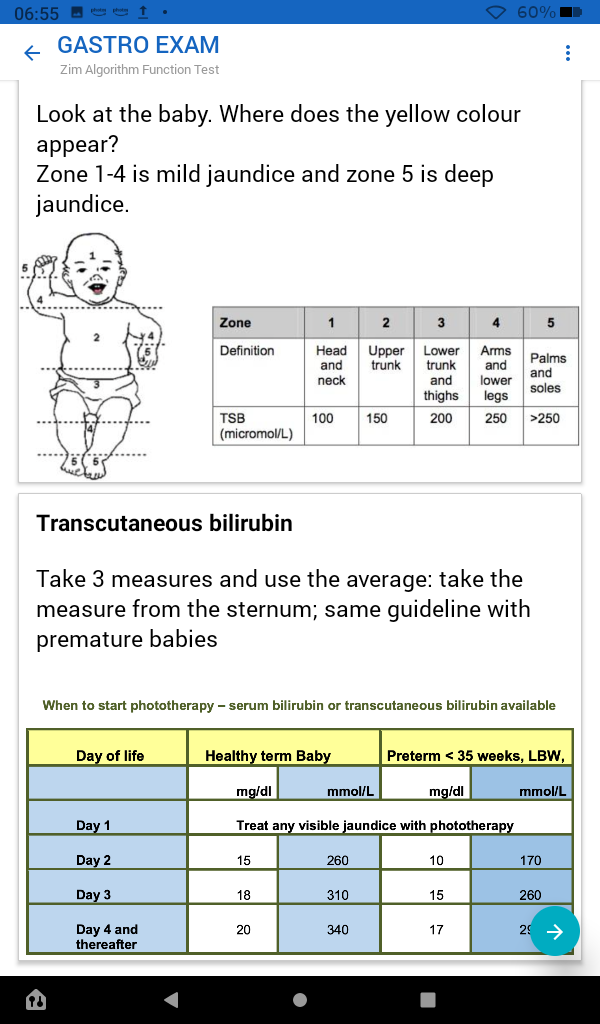


***Figure S4: Example of Jaundice assessment page in the Neotree app***

1. Variable Expression:
2. Pathological jaundice: ($YColour = 'Y' and $Age < 24) or ($YColour = 'Y' and $Gestation =< 35) or ($YColour = 'Y' and ($Jaundice = '5' or $Temperature >= 38 or $BirthWeight < 2500 or $Colour = 'White'))
3. Physiological jaundice: ($Jaundice = '1' or $Jaundice = '2' or $Jaundice = '3' or $Jaundice = '4' )and $Age > 24 and (($Gestation >= 37 and $Age < 336) or ($Gestation < 37 and $Age < 504))
4. Prolonged jaundice: $YColour = 'Y' and (($Age > 336 and $Gestation >= 37) or ($Age > 504 and $Gestation =< 35))
5. Management:

**Physiological jaundice**

| Zimbabwe Management Pages | Malawi Management Pages |
| --- | --- |
| 1. Take Transcutaneous Bilirubin (TCB) measurement  Take 3 TCB measurements from the sternum and plot the average TCB on graph  Take the serum bilirubin as well if possible.  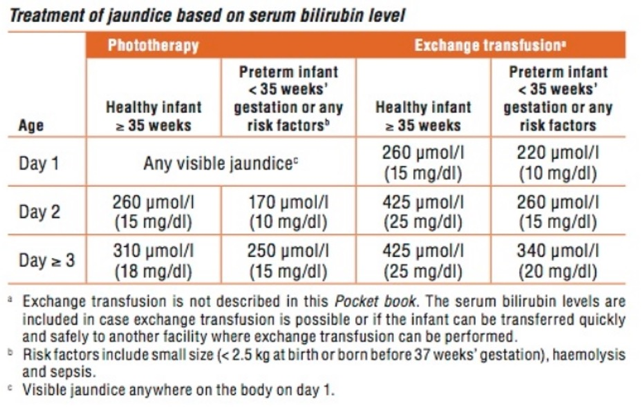 | 1. Take Transcutaneous Bilirubin (TCB) measurement  Take a TCB measurement from the forehead, sternum and abdomen and plot the highest of these 3 measurements  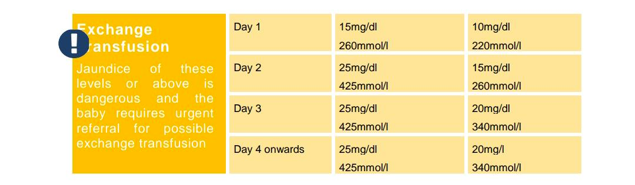  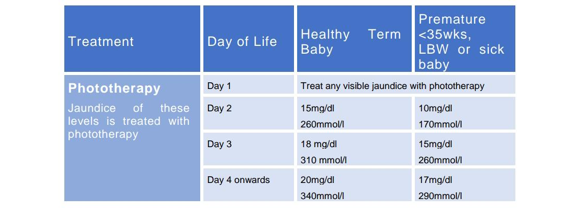 |
| 2. Encourage and support breastfeeding | 2. Encourage and support breastfeeding |
| 3. Educate parents on how to monitor for jaundice | 3. Educate parents on how to monitor for jaundice |

**Pathological jaundice**

| Zimbabwe Management Pages | Malawi Management Pages |
| --- | --- |
| 1. Start phototherapy immediately  Take transcutaneous bilirubin and send serum bilirubin and plot  Send full blood count, blood group (mother and baby) and syphilis serology. Perform coombs test and glucose 6-phosphate deficiency if available.  Take the TSB as well if possible.  Always consider and treat for infection and perform Thompson score to assess for encephalopathy  Start phototherapy  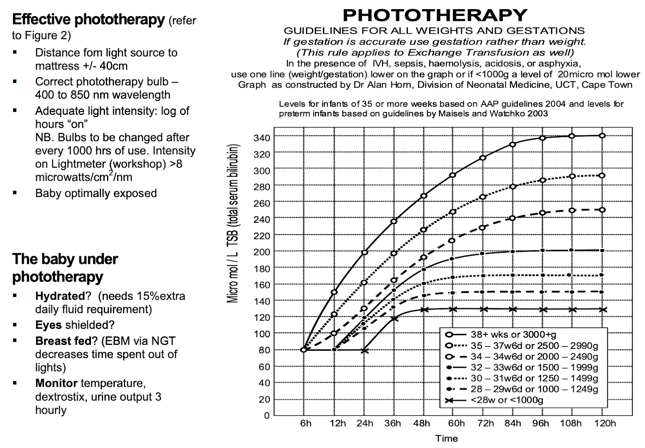 | 1. Start phototherapy immediately  Take transcutaneous bilirubin and send serum bilirubin and plot  Send full blood count, blood group (mother and baby) and syphilis serology. Perform coombs test and glucose 6-phosphate deficiency if available.  Always consider and treat for infection and perform Thompson score to assess for encephalopathy  Start phototherapy  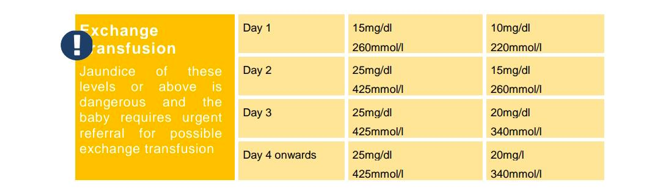  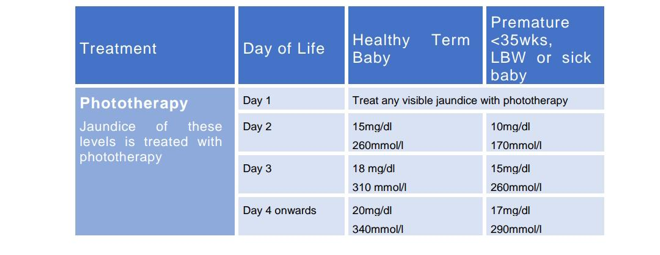 |
| 2. Whilst on phototherapy  Repeat transcutaneous bilirubin every 24 hours/once a day after starting or stopping and plot trend.  Give eye protection for baby  Undress to nappy  Maintain normothermia  Encourage and support breast feeding  Assess for signs of dehydration: Give 20 mL/kg top up with cup EBM or OGT if necessary  Send serum bilirubin if clinically deteriorating  If bilirubin rises above exchange transfusion line, perform exchange transfusion if safe and possible to do so  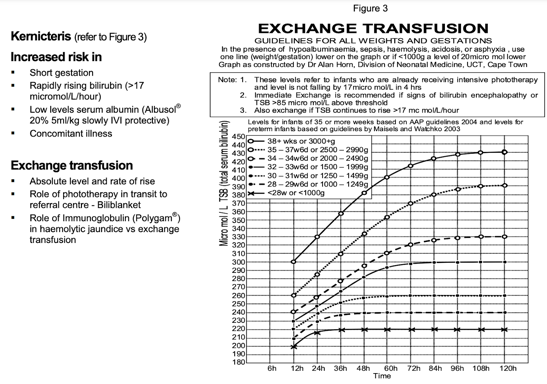 | 2. Whilst on phototherapy  Repeat transcutaneous bilirubin every 24 hours/once a day after starting or stopping and plot trend.  Give eye protection for baby  Undress to nappy  Maintain normothermia  Encourage and support breast feeding  Assess for signs of dehydration: Give 20 mL/kg top up with cup EBM or OGT if necessary  Send serum bilirubin if clinically deteriorating  If bilirubin rises above exchange transfusion line, perform exchange transfusion if safe and possible to do so  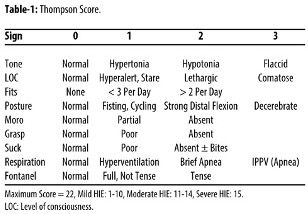 |
| 3. When to stop phototherapy  Stop phototherapy if bilirubin is under the treatment threshold range or no jaundice of palms and soles | 3. When to stop phototherapy  Stop phototherapy if bilirubin is under the treatment threshold range or no jaundice of palms and soles |

**Prolonged jaundice**

| Zimbabwe Management Pages | Malawi Management Pages |
| --- | --- |
| 1. Needs further investigation to exclude:  Liver/metabolic disorder – Perform liver function tests and calculate percentage of conjugated to  unconjugated bilirubin (if more than 20% it is conjugated hyperbilirubinemia)  Hypothyroidism  - perform thyroid function tests if available  Biliary atresia  - perform abdominal USS if conjugated hyperbilirubinemia and pale stools  Take the TSB as well if possible. | 1. Needs further investigation to exclude:  Liver/metabolic disorder – Perform liver function tests and calculate percentage of conjugated to  unconjugated bilirubin (if more than 20% it is conjugated hyperbilirubinemia)  Hypothyroidism  - perform thyroid function tests if available  Biliary atresia  - perform abdominal USS if conjugated hyperbilirubinemia and pale stools |
| 2. Encourage and support breast feeding | 2. Encourage and support breast feeding |

### HIV exposed

1. Measured/recorded:

Based on maternal history

- 1. Has mother had HIV test? Y/N
  2. If Yes ($MatHIVtest = true);
     1. When was this test? (skip if date unknown)
     2. Test during this pregnancy? Y/N/Unknown
     3. What was the Result? (R),Reactive, (NR),Non Reactive (U),Unknown
     4. Is the mother on highly active antiretroviral therapy (HAART)? Y/N
     5. Mother on HAART since when? 1st Trimester or earlier,2nd Trimester,3rd Trimester more than 1 month before delivery, Late-Less than 1 month before delivery, Unknown

1. Categorisation: based on WHO guidelines and local national guidelines [31,35]

High risk HIV exposed neonate:

- “HIV exposed high risk” - Current conditional expression: ($MatHIVtest = true) and ($HIVtestResult = 'R' or $HIVtestResult = 'U') and ($HAART = 'N' or $LengthHAART = 'Late')

A high-risk infant is defined as being born to a mother who:

- Maternal HIV antenatal status is recorded as reactive
- Had a viral load >1000 copies/mL in the 4 weeks before delivery OR
- Received <4 weeks of ART at delivery OR
- Was newly diagnosed with HIV during labour, delivery or postpartum

If the above conditional expression is met then the below advice page is shown:

Advice:

If the answer is no for either of the following, please make sure these are done urgently!

- - 1. Have they received nevirapine (NVP) & Zidovudine (AZT)? Y/N
    2. Have they had a DNA-PCR? Y/N

Low risk HIV exposed neonate

- “HIV exposed low risk” - defined as an infant( ($MatHIVtest = true) and ($HIVtestResult = 'R' or $HIVtestResult = 'U') and ($LengthHAART = '1stTrim' or $LengthHAART = '2ndTrim' or $LengthHAART = '3rdTrim'))
  1. Maternal HIV antenatal status is recorded as reactive
  2. Born to women established on ART with viral suppression <1000 copies/mL

If the above conditional expression is met, then the below advice page is shown:

Advice page: The baby is LOW RISK - Have they received NVP? - Y/N

HIV status of mother is unknown

- 1. If mother has never had an HIV test and requires a test
  2. Conditional expression: $MatHIVtest = false

No risk

- Maternal HIV antenatal status is recorded as unreactive

1. Variable Expression:

- HIV high risk: ($MatHIVtest = true) and ($HIVtestResult = 'R' or $HIVtestResult = 'U') and ($HAART = 'N' or $LengthHAART = 'Late')
- HIV low risk: ($MatHIVtest = true) and ($HIVtestResult = 'R' or $HIVtestResult = 'U') and ($LengthHAART = '1stTrim' or $LengthHAART = '2ndTrim' or $LengthHAART = '3rdTrim'))
- HIV unknown: $MatHIVtest = false

1. Management:

Any baby who is identified as being born from a HIV exposed mother should receive NVP straight after birth according to WHO and COIN guidelines [30,26,35]. If this occurs, the app automatically leads the HCP to an advice page telling them to give NVP, irrespective of whether or not they click HIV exposed as a potential diagnosis at the end.

High risk infants should receive:

- Daily AZT + NVP for 12 weeks if breast fed
- Daily AZT + NVP for 6 weeks if formula fed
- Infants should have a DNA PCR as soon as possible after delivery and again at 6 weeks if birth PCR negative.
- Serology should be tested at 9 and 18 months.

*Low Risk Infant*

- They should receive daily NVP for 6 weeks
- Infants should have a DNA PCR as soon as possible after delivery and again at 6 weeks if birth PCR negative*
- Serology should be tested at 9 and 18 months*

NVP and AZT dose as per table


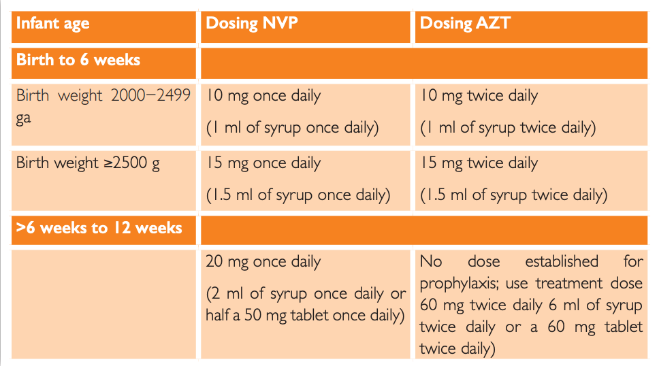


*from 12.2.19; HCPs were advised to carry out a DNA PCR at birth in all HIV exposed babies (i.e. regardless of whether they are categorised as low or high risk) and should be tested again at 6 weeks, and performed serology at 9 and 18 months.

**HIV low risk**

| Zimbabwe Management Pages | Malawi Management Pages |
| --- | --- |
| The baby is LOW RISK | The baby is LOW RISK |
| - These infants are born to women established on ART with viral suppression <1000 copies/mL or more than 4 weeks of antiretroviral therapy (ART)  - They should receive daily NVP for 6 weeks  ALL HIV EXPOSED BABIES SHOULD RECEIVE A DNA PCR AT BIRTH REGARDLESS OF HIGH/LOW RISK  - DNA PCR should be tested again at 6 weeks, and serology at 9 and 18 months.  DNA PCR test: If DNA PCR test positive, commence full ART as soon as possible  Dose as per table | - These infants are born to women established on ART with viral suppression <1000 copies/mL or more than 4 weeks of ART  - They should receive daily NVP for 6 weeks  ALL HIV EXPOSED BABIES SHOULD RECEIVE A DNA PCR AT BIRTH REGARDLESS OF HIGH/LOW RISK  - DNA PCR should be tested again at 6 weeks, and serology at 9 and 18 months.  DNA PCR test: If DNA PCR test positive, commence full ART as soon as possible  Dose as per table |
| 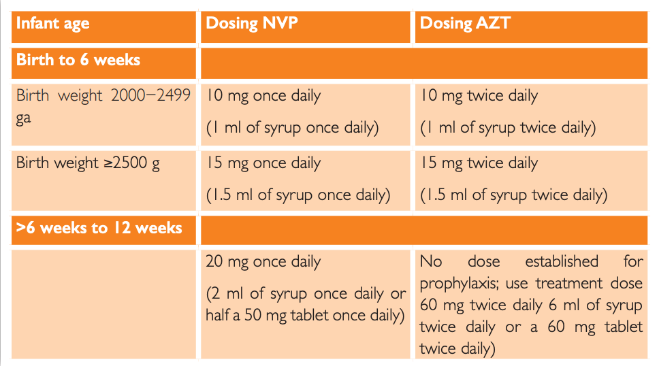 | 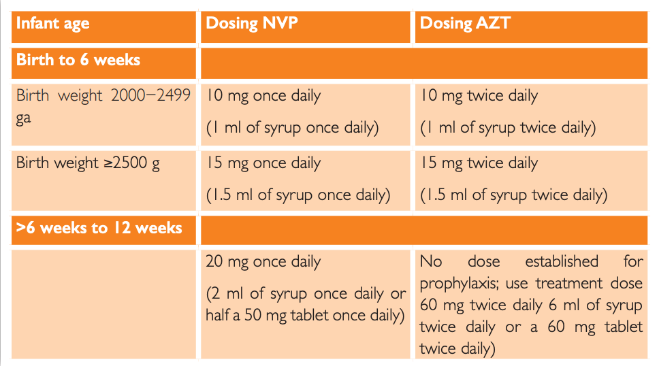 |

**HIV high risk**

| Zimbabwe Management Pages | Malawi Management Pages |
| --- | --- |
| The baby is HIGH RISK!  A high risk infant is born to a mother who:  1. Had a viral load >1000 copies/mL in the 4 weeks before delivery OR  2. Received <4 weeks of ART at delivery OR  3. Was newly diagnosed with HIV during labour, delivery or post partum | The baby is HIGH RISK!  A high risk infant is born to a mother who:  1. Had a viral load >1000 copies/mL in the 4 weeks before delivery OR  2. Received <4 weeks of ART at delivery OR  3. Was newly diagnosed with HIV during labour, delivery or post partum |
| Infants should receive:  Daily AZT + NVP for 12 weeks if breast fed  Daily AZT + NVP for 6 weeks if formula fed  Infants should have a DNA PCR as soon as possible after delivery and again at 6 weeks if birth PCR negative. Serology should be tested at 9 and 18 months.  Dose as per table  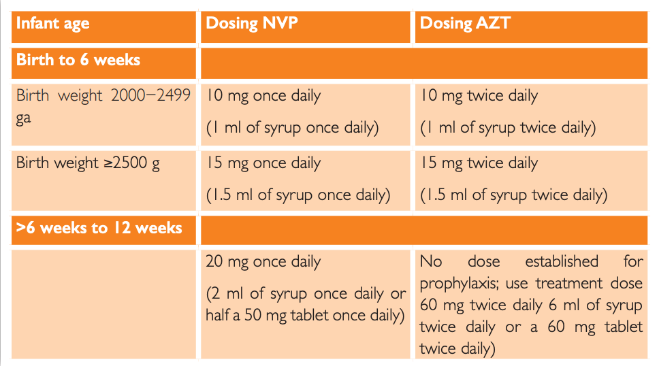 | Infants should receive:  NVP for 12 weeks if breast fed  NVP for 6 weeks if formula fed  Infants should have a DNA PCR as soon as possible after delivery and again at 6 weeks if birth PCR negative. Serology should be tested at 9 and 18 months.  Dose as per table  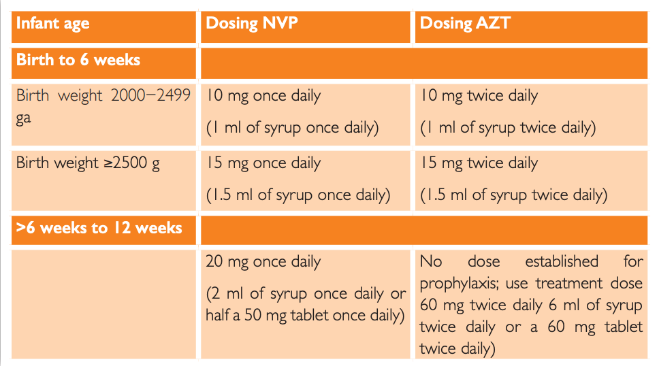 |
| If < 2kg: (as per WHO guidelines)  AZT 4mg /kg per doses 12 hourly  NVP 2mg/kg per day to 2 weeks          4mg/kg per day 2-6 weeks | If < 2kg: (as per WHO guidelines)  NVP 2mg/kg per day to 2 weeks          4mg/kg per day 2-6 weeks |

**HIV unknown**

| Zimbabwe Management Pages | Malawi Management Pages |
| --- | --- |
| This mother and baby need an URGENT HIV test  Rapid HIV test needed and treat as high risk if reactive | This mother and baby need an URGENT HIV test  Rapid HIV test needed and treat as high risk if reactive |
| A high risk infant is born to a mother who:  1. Had a viral load >1000 copies/mL in the 4 weeks before delivery OR  2. Received <4 weeks of ART at delivery OR  3. Was newly diagnosed with HIV during labour, delivery or post partum  Infants should receive:  Daily AZT + NVP for 12 weeks if breast fed  Daily AZT + NVP for 6 weeks if formula fed  Infants should have a DNA PCR as soon as possible after delivery and again at 6 weeks if birth PCR negative. Serology should be tested at 9 and 18 months.  Dose as per table  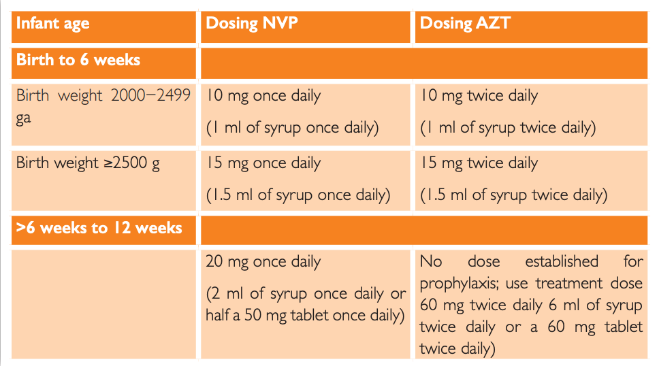 | A high risk infant is born to a mother who:  1. Had a viral load >1000 copies/mL in the 4 weeks before delivery OR  2. Received <4 weeks of ART at delivery OR  3. Was newly diagnosed with HIV during labour, delivery or post partum  Infants should receive:  NVP for 12 weeks if breast fed  NVP for 6 weeks if formula fed  Infants should have a DNA PCR as soon as possible after delivery and again at 6 weeks if birth PCR negative. Serology should be tested at 9 and 18 months.  Dose as per table  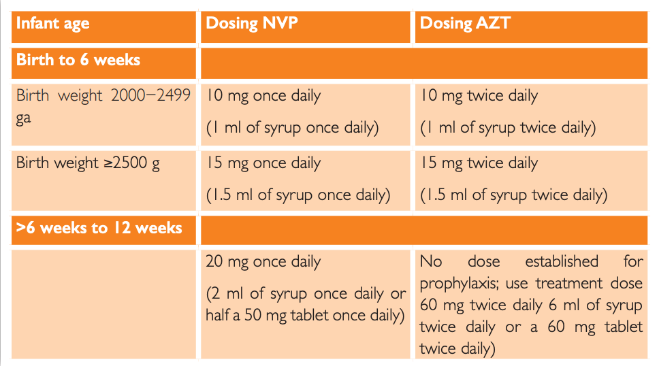 |
| If < 2kg: (as per WHO guidelines)  AZT 4mg /kg per doses 12 hourly  NVP 2mg/kg per day to 2 weeks          4mg/kg per day 2-6 weeks | If < 2kg: (as per WHO guidelines)  NVP 2mg/kg per day to 2 weeks          4mg/kg per day 2-6 weeks |

### Congenital abnormalities

1. Measured/recorded: based on reason for admission and clinical finding

This algorithm is based on any point in the admission form when a congenital abnormality can be picked up, i.e., under admission reason, or during any part of the examination.

1. Categorisation:

Gastroschisis

Omphalocele

Moderate Talipes (club foot)

Mild Talipes (club foot)

Myelomeningocele

Cleft lip

Cleft lip and/or palate

Cleft lip and/or palate with RD

Congenital Dislocation of the Hip (CDH)

1. Variable Expression:

- Cleft lip: ​​$Palate = 'Lip'
- Cleft lip and/or palate: $Palate = 'Cleft'
- Cleft lip and/or palate with RD: ($Palate = 'Lip' or $Palate = 'Cleft' or $Palate = 'LipPalate') and ($RR > 60 or $DangerSigns = 'Grun' or $DangerSigns = 'Cyan' or $SatsAIr < 90 or $SatsO2 < 90 or $RespSR = 'DIB' or $RespSR = 'BE' or $RespSR = 'Apn' or $RespSR = 'Cough' or $RespSR = 'NBr' or $SignsRD = 'TT' or $SignsRD = 'HN' or $SignsRD = 'ST' or $SignsRD = 'NFL' or $SignsRD = 'CHI' or $SignsRD = 'GR' or $SignsRD = 'Gasp' or $SignsRD = 'GR')
- Myelomeningocele: $Spine = 'NTD'
- Congenital Dislocation of the Hip (CDH): $Presentation = 'Breech' or $MatComorbidities = 'CDH' or $MSKproblems = 'Legs' or $Skin = 'Folds' or $Ortolani = 'Yes'
- Moderate Talipes (club foot): $TalipesSev = 'No'
- Mild Talipes (club foot): $TalipesSev = 'Yes'
- Omphalocele: $GSCvsOM = 'OMPH'
- Gastroschisis: $GSCvsOM = 'GSCH'

1. Management:

**Gastroschisis**

| Zimbabwe Management Pages | Malawi Management Pages |
| --- | --- |
| 1. Surgical assessment  Primary repair is considered on day 1 if infant is less than 6 hours old and all bowel is viable.  If there are signs of sepsis or non-viable bowel (conduct PEEL Score) then surgeons apply a silo bag  If it’s viable and <6 hours the surgeons do primary repair on day 1. If there are signs of sepsis or distended bowels, then you add a silo bag.  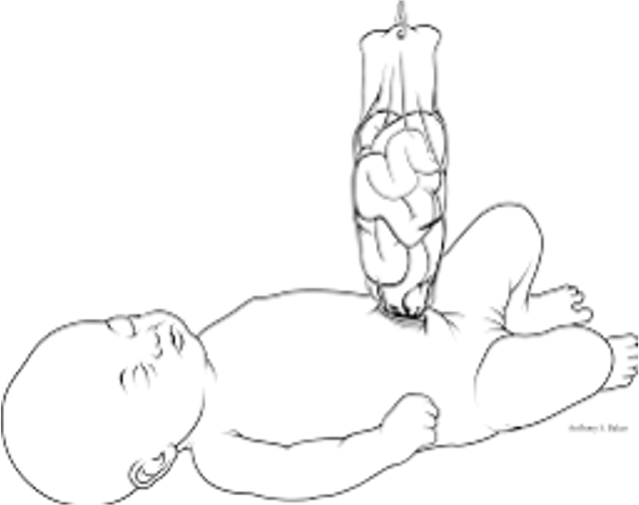 | 1. Surgical assessment  Primary repair is considered on day 1 if infant is less than 6 hours old and all bowel is viable.  If there are signs of sepsis or non-viable bowel (conduct PILO SCORE) then surgeons apply a silo bag  If it’s viable and <6 hours the surgeons do primary repair on day 1. If there are signs of sepsis or distended bowels, then you add a bag.  If it’s viable and <6 hours the surgeons do primary repair on day 1. If there are signs of sepsis or distended bowels, then you add a bag.  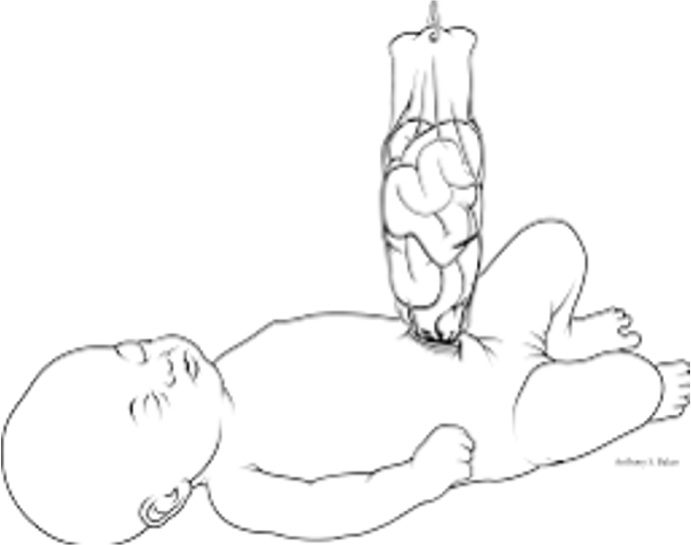 |
| 2. Paediatrician to review for other congenital abnormalities | 2. Paediatrician to review for other congenital abnormalities |
| 3. Antibiotics  If possible, conduct a blood culture and then start triple antibiotics  Ampicillin (25-50mg/kg IV four times a day) and  Amikacin (7.5mg/kg IV once a day) and  Metronidazole (15mg/kg as a single loading dose, followed by 7.5mg/kg every 12hr starting 24 hr after the loading dose)  4. Counsel and educate parents  Encourage parental bonding | 3. Antibiotics  Start triple antibiotics  Ampicillin (25-50mg/kg IV four times a day) and  Gentamicin (7.5mg/kg IV once a day) and  Metronidazole (15mg/kg as a single loading dose, followed by 7.5mg/kg every 12hr starting 24 hr after the loading dose)  4. Counsel and educate parents  Encourage parental bonding |

**Omphalocele**

| Zimbabwe Management Pages | Malawi Management Pages |
| --- | --- |
| 1. Surgical assessment  Gentian violet paint applied | 1. Surgical assessment  Gentian violet paint applied |
| 2. Paediatrician to review for other congenital abnormalities | 2. Paediatrician to review for other congenital abnormalities |
| 3. Antibiotics  Start triple antibiotics  Ampicillin (25-50mg/kg IV four times a day) and  Gentamicin (7.5mg/kg IV once a day) and  Metronidazole (15mg/kg as a single loading dose, followed by 7.5mg/kg every 12hr starting 24 hr after the loading dose)  4. Counsel and educate parents  Encourage parental bonding | 3. Antibiotics  Start triple antibiotics  Ampicillin (25-50mg/kg IV four times a day) and  Gentamicin (7.5mg/kg IV once a day) and  Metronidazole (15mg/kg as a single loading dose, followed by 7.5mg/kg every 12hr starting 24 hr after the loading dose)  4. Counsel and educate parents  Encourage parental bonding |

**Moderate Talipes**

| Zimbabwe Management Pages | Malawi Management Pages |
| --- | --- |
| 1. Educate and counsel parents needs immediate attention  Refer to physiotherapist or rehabilitation technician | 1. Educate and counsel parents needs immediate attention  Refer to physiotherapist or rehabilitation technician |
| 2. Serial manipulations beginning shortly after birth (see pictures).  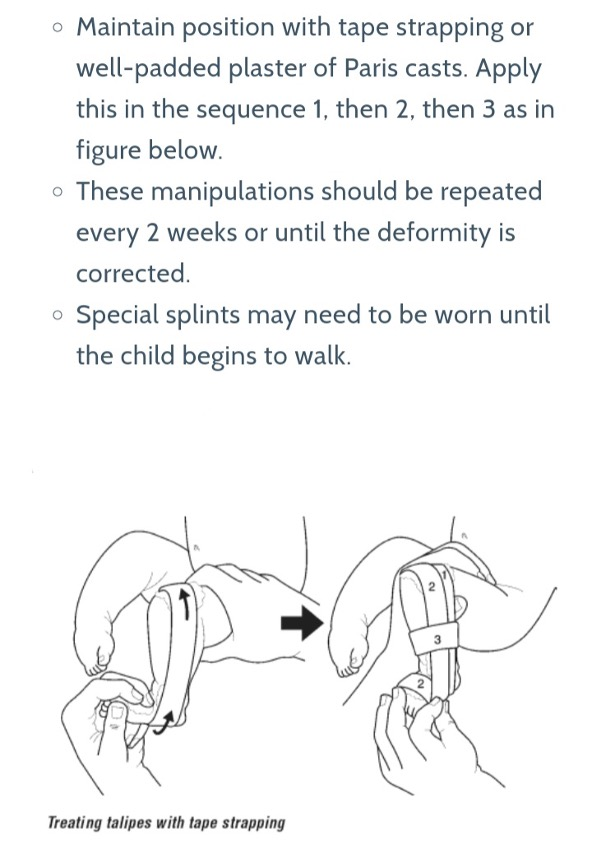 | 2. Serial manipulations beginning shortly after birth (see pictures).  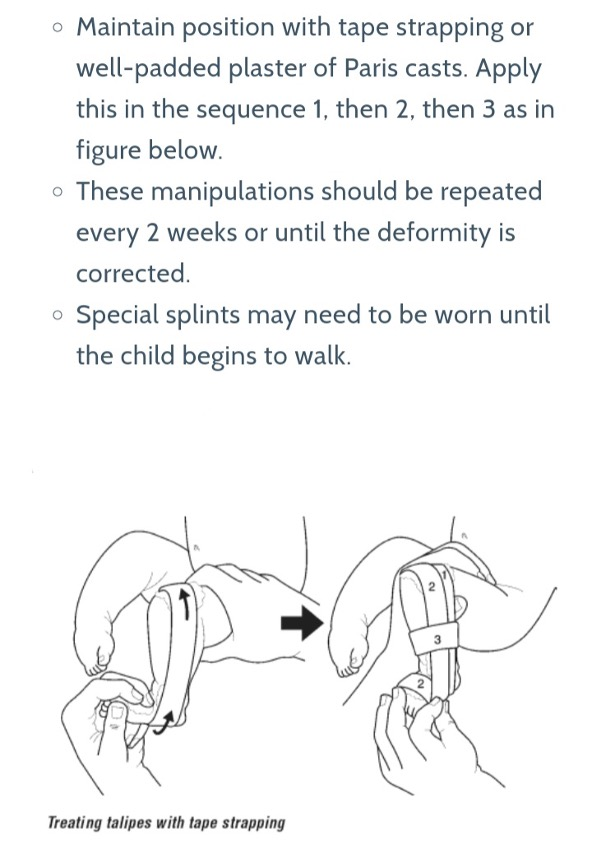 |
| 3. Severe deformity or late presentation requires surgical repair | 3. Severe deformity or late presentation requires surgical repair |

**Mild Talipes**

| Zimbabwe Management Pages | Malawi Management Pages |
| --- | --- |
| 1. Educate and counsel parents needs immediate attention  Refer to physiotherapist or rehabilitation technician | 1. Educate and counsel parents needs immediate attention  Refer to physiotherapist or rehabilitation technician |
| 2. Simple stretching of foot beginning shortly after birth | 2. Simple stretching of foot beginning shortly after birth |

**Myelomeningocele**

| Zimbabwe Management Pages | Malawi Management Pages |
| --- | --- |
| 1. Handle with sterile, latex-free gloves (if possible) and sterile towels at delivery  Nurse side-lying or prone to ensure no pressure is placed on the lesion  Wrap lesion with sterile gauze soaked in warm saline | 1. Handle with sterile, latex-free gloves (if possible) and sterile towels at delivery  Nurse side-lying or prone to ensure no pressure is placed on the lesion  Wrap lesion with sterile gauze soaked in warm saline |
| 2. Antibiotics  Apply topical tetracycline and give IV Benzylpenicillin (100-150 mg/kg daily in two divided doses) or Ampicillin (25-50 mg/kg IM or IV four times a day) PLUS Amikacin for 5 days  Refer to Paediatric Neurosurgeon for closure. | 2. Antibiotics  Apply topical tetracycline and give IV Benzylpenicillin (100-150 mg/kg daily in two divided doses) or Ampicillin (25-50 mg/kg IM or IV four times a day) PLUS Amikacin for 5 days  Refer to Paediatric Neurosurgeon for closure. |
| 3. Monitoring  Monitor daily head circumference  and obtain Cranial ultrasound (USS) and CT (if CrUSS is of concern and possible)  Monitor for symptoms of bowel, bladder and motor deficit and hydrocephalus (apnoea, bradycardia, hypoventilation, stridor, swallow dysfunction)  Monitor urine output and obtain renal ultrasound (if possible). Check Creatine Day 5 to 7 | 3. Monitoring  Monitor daily head circumference  and obtain Cranial USS and CT (if CrUSS is of concern and possible)  Monitor for symptoms of bowel, bladder and motor deficit and hydrocephalus (apnoea, bradycardia, hypoventilation, stridor, swallow dysfunction)  Monitor urine output and obtain renal ultrasound (if possible). Check Creatine Day 5 to 7 |

**Cleft lip**

| Zimbabwe Management Pages | Malawi Management Pages |
| --- | --- |
| 1. Feeding  Feed normally  Monitor feeding and growth, may need cup or spoon top up feeds | 1. Feeding  Feed normally  Monitor feeding and growth, may need cup or spoon top up feeds |
| 2. Review by Paediatrician for other congenital abnormalities.  Refer to maxillofacial surgeons, usually repaired at 6 months. | 2. Review by Paediatrician for other congenital abnormalities.  Refer to surgeons, usually repaired at 6 months. |
| 3. Counsel and educate  Encourage maternal and parental bonding | 3. Counsel and educate  Encourage maternal and parental bonding |

**Cleft lip and/or palate**

| Zimbabwe Management Pages | Malawi Management Pages |
| --- | --- |
| 1. Feeding  Give expressed breast milk (EBM) via cup or spoon or special teat  STOP feeding if any signs of aspiration or gagging  Give small feeds 15-20mLs at a time and burp well  Monitor vital signs every 2 hours  Close monitoring of feeding and growth | 1. Feeding  Give expressed breast milk (EBM) via cup or spoon or special teat  STOP feeding if any signs of aspiration or gagging  Give small feeds 15-20mLs at a time and burp well  Monitor vital signs every 2 hours  Close monitoring of feeding and growth |
| 2. Referrals  Review by Paediatrician for other congenital abnormalities.  Refer to maxillofacial surgeons – usually repaired at 1 year of age  Follow up after surgery to monitor hearing and speech | 2. Referrals  Review by Paediatrician for other congenital abnormalities.  Refer to surgeons – usually repaired at 1 year of age  Follow up after surgery to monitor hearing and speech |
| 3. Counsel and educate  Encourage maternal and parental bonding | 3. Counsel and educate  Encourage maternal and parental bonding |

**Cleft lip and/or palate with RD**

| Zimbabwe Management Pages | Malawi Management Pages |
| --- | --- |
| 1. Feeding  Nasogastric feeds with EBM (plus non-nutritive breast feeds for less than 10 minutes at a time) | 1. Feeding  Nasogastric feeds with EBM (plus non-nutritive breast feeds for less than 10 minutes at a time) |
| 2. Referrals  Review by Paediatrician for other congenital abnormalities.  Refer to maxillofacial surgeons – usually repaired at 1 year of age  Follow up after surgery to monitor hearing and speech | 2. Referrals  Review by Paediatrician for other congenital abnormalities.  Refer to surgeons – usually repaired at 1 year of age  Follow up after surgery to monitor hearing and speech |
| 3. Counsel and educate  Encourage maternal and parental bonding | 3. Counsel and educate  Encourage maternal and parental bonding |

**Congenital Dislocation of the Hip (CDH)**

| Zimbabwe Management Pages | Malawi Management Pages |
| --- | --- |
| Consider congenital dislocation of the hip for the baby for the following reasons. Either:  1. Presentation of the baby is breech  2. Family history of CDH  3. Legs are of different length  4. Skin folds on legs  5. Clunks/clicks during the Ortolani manoeuvre or Barlow's test | Consider congenital dislocation of the hip for the baby for the following reasons. Either:  1. Presentation of the baby is breech  2. Family history of CDH  3. Legs are of different length  4. Skin folds on legs  5. Clunks/clicks during the Ortolani manoeuvre or Barlow's test |
| 2. Referrals  Review by neonatal unit (NNU) Consultant for ongoing follow-up | 2. Referrals  Review by NNU Consultant for ongoing follow-up |
